# Nucleus segmentation across imaging experiments: the 2018 Data Science Bowl

**DOI:** 10.1038/s41592-019-0612-7

**Published:** 2019-10-21

**Authors:** Juan C. Caicedo, Allen Goodman, Kyle W. Karhohs, Beth A. Cimini, Jeanelle Ackerman, Marzieh Haghighi, CherKeng Heng, Tim Becker, Minh Doan, Claire McQuin, Mohammad Rohban, Shantanu Singh, Anne E. Carpenter

**Affiliations:** Broad Institute of MIT and Harvard, Cambridge, MA USA

**Keywords:** Machine learning, Image processing

## Abstract

Segmenting the nuclei of cells in microscopy images is often the first step in the quantitative analysis of imaging data for biological and biomedical applications. Many bioimage analysis tools can segment nuclei in images but need to be selected and configured for every experiment. The 2018 Data Science Bowl attracted 3,891 teams worldwide to make the first attempt to build a segmentation method that could be applied to any two-dimensional light microscopy image of stained nuclei across experiments, with no human interaction. Top participants in the challenge succeeded in this task, developing deep-learning-based models that identified cell nuclei across many image types and experimental conditions without the need to manually adjust segmentation parameters. This represents an important step toward configuration-free bioimage analysis software tools.

## Abstract (web summary)

The 2018 Data Science Bowl challenged competitors to develop an accurate tool for segmenting stained nuclei from diverse light microscopy images. The winners deployed innovative deep-learning strategies to realize configuration-free segmentation.

## Main

Microscopy is a central technology of biomedical research, mentioned in nearly one million PubMed-indexed scientific papers to date (PubMed search ‘microscopy OR microscope OR microscopic’, accessed 7 October 2018). Increasingly, the images produced are analyzed quantitatively^1–3^. Various microscopy techniques allow capturing structural and functional properties of biological model systems, including cultured cells, tissues and organoids. As microscopy makes progress to capture such systems in greater detail and throughput and as the development of novel assays reveals more complex properties of living organisms, the need for robust and easy to use microscopy image analysis methods becomes critical to answer a wider variety of biological questions.

Many image analysis workflows involve the identification (segmentation) of cell nuclei as a first step to extract meaningful biological signals. Research studies may involve counting cells, tracking moving populations, localizing proteins and classifying phenotypes or profiling treatments; in all of these and more, the nucleus is a reliable compartment of reference for identifying single cells in microscopy images.

However, selecting strategies to segment nuclei is not an easy task for nonexpert users in regular biology labs. Most existing user-friendly bioimage analysis tools^4–6^ identify nuclei using classical segmentation algorithms such as thresholding^7^, watershed^8^ or active contours^9^. These need to be configured for each study to account for different microscopy modalities, scales and experimental conditions, often requiring great expertise to select the algorithm that suits the problem and to adjust its parameters. For advanced users, the choice can also be daunting, considering that hundreds of papers are published every year presenting new methods for cell and nucleus segmentation. And even under controlled experimental conditions, no single parameter choice can segment all images correctly, because classical algorithms can fail to adapt to the heterogeneity of biological samples or can be sensitive to technical artifacts^10–12^. Altogether, this situation slows down the pace of research and hinders biological laboratories from adopting imaging technologies owing to the time and expertise required.

Here, we explore the idea of creating a segmentation model that can identify the nucleus of cells automatically in a diverse set of stained two-dimensional (2D) light microscopy images without human interaction. Such a model could power future robotic microscopes to facilitate a wide range of biological applications, by finding and counting nuclei in images in real time across cell types, staining types, magnification and in spite of experimental variations. A single trained model effective across instruments, stains and cell types would improve the experience of biologists and speed their research. Classical algorithms for identifying nuclei in microscopy images follow very similar computational strategies, with varying parameters or configurations (Methods, Supplementary Note 3). Our goal was to investigate whether any modern solutions, such as large capacity deep-learning models, could provide a single unifying solution without requiring manual configuration.

Biological image segmentation on the basis of machine learning already exists in user-friendly software, such as Ilastik^13^ and ImageJ^14^, and recent studies confirm the usefulness of this approach^15^. Deep learning has shown great potential to solve difficult problems in cellular image analysis^16^, and neural network models for image segmentation also exist^17–20^. However, existing solutions require users to create models that are customized for each experiment, taking time to prepare annotations, train models and/or configure algorithms. We instead aimed to create a generic, reusable model that is trained once and can be shared and run on a variety of fluorescence microscopy experiments without additional user intervention. We envisioned software tools for nucleus segmentation that can be used with the same ease and robustness as face detectors in natural images; they just work, without users having to train models or to configure settings and under varying lighting and scenery conditions.

This paper reports the results of the 2018 Data Science Bowl, which challenged participants to segment nuclei in a variety of 2D light microscopy images without the need for any manual interaction or adjustment. The competition provided participants with a training set of images comprising problems (images containing nuclei) along with the corresponding solutions (segmentation masks for the nuclei) and test sets of images for which they had to generate the segmentations using a two-stage evaluation protocol. Importantly, the holdout set was comprised of 15 diverse image sets from biological experiments that were not present in the training set, to realistically evaluate how well the algorithms perform across different experimental conditions. This is the first time that nucleus segmentation methods have been challenged to generalize by operating blindly on unseen biological experiments without user interaction or additional annotation/training.

## Results

### The 2018 Data Science Bowl

For the competition, we created a dataset with 37,333 manually annotated nuclei in 841 2D images from more than 30 experiments across different samples, cell lines, microscopy instruments, imaging conditions, operators, research facilities and staining protocols. The annotations were manually made by a team of expert biologists that followed a collaborative workflow (Methods), and we call these ‘target masks’ instead of ground truth, given that each annotation was created by a single expert and reviewed by the rest. Researchers around the world freely contributed the images and agreed to a Creative Commons 0 license (public domain); our team’s annotations are similarly freely available. This dataset is publicly accessible in the Broad Bioimage Benchmark Collection with accession number BBBC038.

The challenge was run for a total of 3 months in which participants had access to the training set (with target masks) and the first-stage test sets (with target masks withheld). The evaluation of competitors’ predictions on the withheld masks of the first-stage test set powered the leaderboard, and in the final week of the competition, a second-stage test set (with target masks withheld) was released to determine the challenge winners. This second-stage evaluation aimed to assess the robustness of models to segment new images from new experiments and to also evaluate the ability of the models to run completely autonomous segmentation without user interaction. To deter manual intervention on the images, the second-stage holdout set had 3,200 images with approximately 100,000 single nuclei that had to be segmented in <7 d, only a small fraction of which had accompanying manually defined target masks and were actually used for scoring (Methods). Participants uploaded their segmentation masks to the Kaggle server (https://www.kaggle.com/c/data-science-bowl-2018), which validated against the real masks hidden from the public, using a quantitative score to rank participants (Methods).

A total of 17,929 competitors signed up for the competition in 3,891 teams during the first stage and 739 teams made successful entries during the second stage to compete for US$170,000 in cash and prizes. Overall, participants submitted a total of 68,017 submissions throughout the duration of the competition. This contest fostered the development of new methods with contributions of data scientists around the world that usually do not work on microscopy images, bringing state-of-the-art innovations. The top three participants, among many other competitors in the challenge, made their solutions open source, which will facilitate their adoption and extension by the wider scientific community.

### Top solutions improve usability and accuracy of nucleus segmentation

In the second-stage evaluation, competitors were not permitted to use any nonautomated, image-specific configuration. As a result, the winning models yielded a major improvement in usability compared to current practices for microscopy image segmentation, which need either algorithm selection and tuning (for classical methods) or manual annotations (for machine-learning methods) for different image sets.

**Fig. 1:**
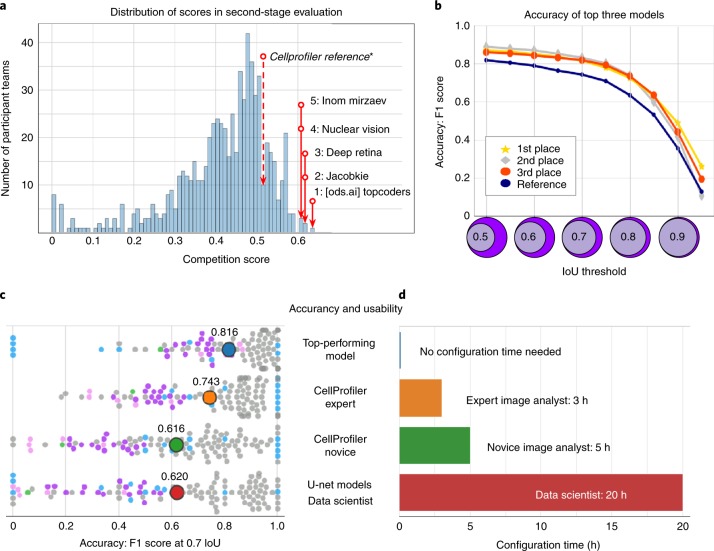
Accuracy and usability of segmentation strategies in the second-stage holdout sets. **a**, The histogram counts participant teams (*n* = 739) according to the official competition score of their best submission. The top five competitors are labeled in the distribution, as is the reference segmentation obtained by an expert analyst using CellProfiler. **b**, Accuracy of the top three solutions measured as the F1 score at multiple IoU thresholds. The scale of the *x* axis of the histogram in panel **a** (competition score) is correlated with the area under the curve of the F1 score versus IoU thresholds. The top three models had a similar performance with slight differences at the tails of the curves. **c**, Breakdown of accuracy in the second-stage evaluation set for the top performing model and three reference solutions. The distribution of F1-scores at a single IoU threshold (IoU = 0.7) shows points (*n* = 106) that each represented the segmentation accuracy of one image in the set of 106 annotated images of the second-stage evaluation (Methods). The color of single-image points corresponds to the group of images defined for reference evaluations (Methods and Fig. 2). The average of the distribution is marked with a larger point labeled with the corresponding average accuracy value. **d**, Estimated time required to configure the segmentation tools evaluated in **c** (Supplementary Note 4).

When compared to a reference segmentation obtained with classical image processing techniques adapted for the holdout image sets using minimal user intervention (Methods), we found that 85 candidate algorithms from the challenge yielded higher accuracy (Fig. 1a). In particular, the top three solutions outperformed these minimally tuned classical algorithms by a large margin, producing better segmentations over all coverage thresholds (Fig. 1b). We evaluated the accuracy of the segmentations using metrics common in computer vision research for object segmentation (Methods).

Importantly, the segmentations obtained with classical image processing algorithms, unlike the methods in the challenge, required manual configuration; there exists no classical algorithm that could claim to produce reasonable results on 15 diverse image sets with no user intervention. In contrast, a novice with no prior exposure to bioimage analysis performed substantially worse than the expert and the top-scoring deep-learning models (Fig. 1c). The novice and expert invested 5 h and 3 h of work to achieve their corresponding segmentation results. Embedded in a user-friendly interface, as has already been prototyped in the NucleAIzer system^21^, the top models would require no configuration time.

The classical methods were tested by first organizing images into five groups using visual inspection, then analysis pipelines were created in the open source software, CellProfiler^5^ (Methods). These pipelines are representative of widely adopted techniques surveyed recently in the literature of microscopy image segmentation^12,22,23^, though they are likely suboptimal solutions because the techniques were not fully optimized for each of the 15 image sets independently to prevent overfitting and in keeping with the no- or low-configuration mission of the data challenge.

In addition to reference segmentations using classical techniques, we also evaluated the performance of the top-scoring models to deep-learning models trained separately for each type of images. We chose U-Net^17^, a popular deep-learning-based method to solve microscopy image segmentation problems, including nucleus segmentation^20^. The learning capacity of a single regular U-Net was not sufficient to capture the experimental variation in the challenge (such solutions entered the competition); therefore, we trained five models to reduce variance, as in the case of classical algorithms, and applied image pre and post-processing routines specific to each group (Supplementary Note 4). The results show that even spending ~20 h of hands-on time (development and training time not included in our estimates), these models did not reach competitive performance compared to the top solution (Fig. 1c). Several factors contributed to this result: limited learning capacity of the evaluated U-Net relative to the top models, reduced number of training examples in the five groups after splitting and experimental variability of the test sets.

Finally, we asked an additional annotator to create target masks for a subset of images in the test set and we observed inter-observer variability (Supplementary Fig. 3) a well-known problem^24^. Interestingly, the top performing model agreed on boundary annotations more often with each annotator than they agreed with each other (Supplementary Fig. 3). This suggests that the model fitted smooth boundaries that were close to the edge of nuclei, whereas manual annotations may be biased by subjective noise (Supplementary Fig. 5). The performance of the top model was also more similar to humans than to classical algorithms in terms of segmentation accuracy (Supplementary Fig. 3). Although we did not investigate this result extensively, it suggests that the top models may reach human-annotator-like performance with similar error rates.

### Best-performing solutions segment a diversity of microscopy images

**Fig. 2:**
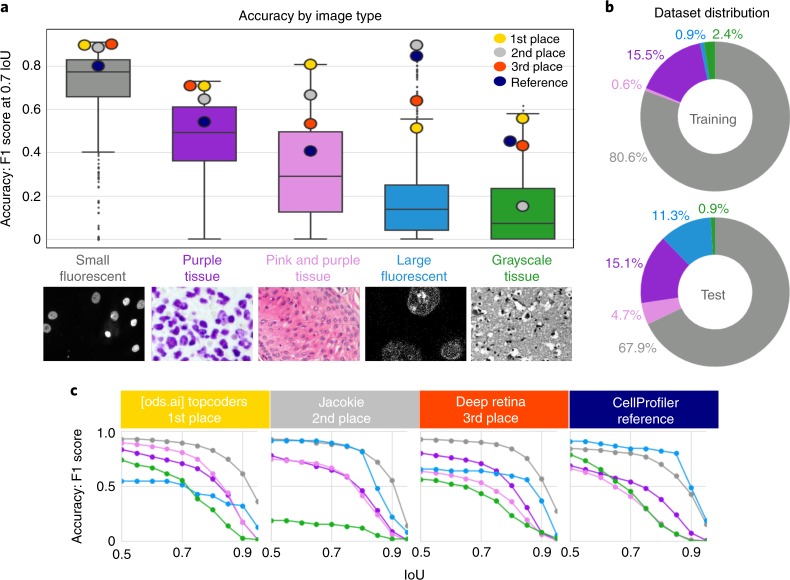
Performance of submitted solutions across varying, imbalanced image types. **a**, Example images of the five visually grouped image types (Methods) are shown across the bottom and the chart shows the spread of F1 scores (Methods) across all second-stage submissions. F1 scores were measured at a threshold of 0.7 IoU (Methods). Box plot: center line, median; box limits, upper and lower quartiles; whiskers, 1.5× interquartile range; points, outliers; colored points, top three participants. **b**, The distribution of the various image types is shown, color-coded as in **a**. The top competitors segmented all image types with high accuracy despite the imbalance of examples in the training set. **c**, Detail of accuracy results by image types and object coverage (IoU) thresholds. The *x* axis displays IoU thresholds and the *y* axis represents accuracy measured with F1 scores. For each participant, the plot displays five curves showing the trend of segmentation accuracy at different object coverage thresholds.

The most challenging aspect of the competition was that the holdout set included microscopy images from 15 different biological experiments, including various 2D light microscopy types, acquisition equipment and biological conditions. This is in contrast to previous research studies that optimize nucleus segmentation methods individually for each image set or type^12,18,22,25–28^. From a visual standpoint, we identified five groups of images comprising nuclei of very different appearances, including two major types of light microscopy (Fig. 2a): fluorescence microscopy of mainly cultured cells and brightfield microscopy of stained tissue samples. Tissue samples are typically a more challenging image processing task owing to the irregular appearances of nuclei and their crowded layout. Small fluorescent nuclei images are very common in biomedical research and the most common in both training and test sets. The entire dataset included 31 different experiments (16 for training and first-stage evaluation, 15 for second-stage evaluation), representing 22 cell types, 15 image resolutions and five groups of visually similar images, resulting in 841 images and 37,333 manually annotated nuclei (Methods). We omitted dramatically different modalities such as unstained brightfield microscopy and electron microscopy.

Top participants stood out by making models that generalized well across diverse image types and experimental variation (Fig. 2a), and despite a heavily unbalanced dataset (Fig. 2b). Dataset biases can mislead the performance of machine-learning models^29,30^; less-represented image types were indeed challenging to segment for the average participant (Fig. 2a). With the largest group of images, containing 80% of the training examples, top participants reached a maximum accuracy of 0.90 in the test set, and with the smallest group, containing 0.6% of the examples, they reached a maximum accuracy of 0.55. In all cases, their performance surpassed the reference CellProfiler segmentations, as well as the average participant, by a large margin.

### Best-performing solutions reduce segmentation errors

**Fig. 3:**
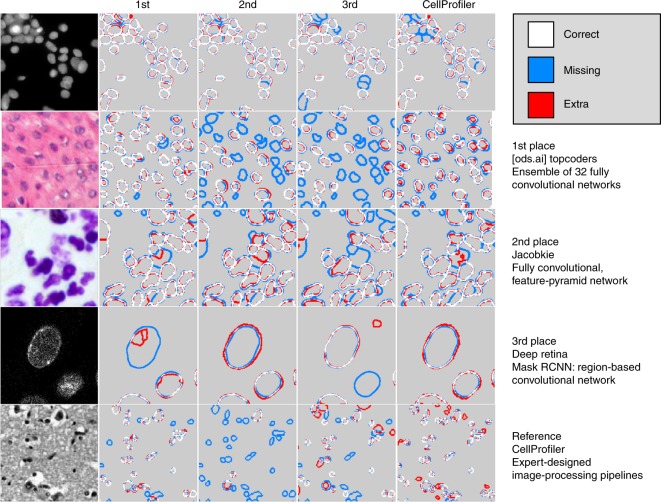
Example segmentation maps for various images obtained by the top three participants and the CellProfiler reference. The segmentation maps show pixel-wise alignments between target segmentation masks and predicted segmentations. If the masks align correctly, pixels in the boundaries are colored white. If the target mask or part of it is missing, pixels in the boundaries are colored blue. If the predicted segmentation is introduced in a region without real object, the boundary pixels are red.

The solutions of the top three teams were significantly better than the reference segmentations according to the competition score and other metrics that we used to analyze the results (Figs. 1–3 and Supplementary Figs. 3 and 4). The competition score was an aggregated metric that considered multiple factors of segmentation quality, including precision, recall and object coverage (Methods) and could be deconstructed in multiple ways to understand performance and error modes.

First, we assessed performance at a single-object coverage threshold to interpret the differences in accuracy between the models. When a threshold equal to 0.5 was chosen (common in previous works^12,31^), the top performing model got an F1 accuracy of 0.889, compared to 0.819 for the CellProfiler reference (Supplementary Table 3). Note that these results were the average across all images from 15 different experiments in the second-stage evaluation, 12 cell lines and five image types. When we considered fluorescent images with only small nuclei, the F1 accuracy of the top performing model was 0.932, whereas CellProfiler obtained an F1 score of 0.844 (Supplementary Table 2). Using a threshold of 0.7 (Fig. 2a) challenges methods by requiring a larger minimum object coverage. We observed that the top three models all surpassed the CellProfiler reference for three image types (small fluorescent, purple and pink and purple tissues). For the other two image types (big fluorescent and grayscale tissue), all but one model performed worse than the CellProfiler reference, primarily owing to the limited number of examples in the training set (Fig. 2).

In general, the top three models reached similar aggregated performance, but exhibited different behavior and error modes. The accuracy results can be disaggregated by image type across multiple object coverage thresholds (Fig. 2c), allowing us to identify the strengths and weaknesses of each strategy. For instance, the second place solution was the best for large fluorescent nuclei (blue line) but poorer for grayscale tissue (green line).

We conducted other performance analyses on the top three models relative to the CellProfiler reference (Supplementary Note 6) and observed reduced error rates from the models, which missed fewer objects, successfully separated merged nuclei (Supplementary Fig. 6) and improved precision and recall (Supplementary Fig. 7). All these observations support the idea that the top three solutions can make configuration-free segmentation of 2D stained nuclei a reality.

### Top algorithms were on the basis of deep convolutional neural networks

The majority of participants used deep convolutional neural networks (CNNs), a popular technique to solve computer vision tasks^32^, as well as various microscopy image and pathology problems^16,26^. A wide variety of CNN architectures can be used for image segmentation and participants designed creative solutions to improve segmentation accuracy. Interestingly, the top three participants used very different solutions: an ensemble of U-Nets, a fully convolutional feature pyramid network (FPN) and a Mask-RCNN (region-based CNN) model. Their performance is summarized in Table 1 and the main characteristics of each model are described below and in the Methods. Figure 3 presents example segmentations obtained by these models together with a reference segmentation obtained by CellProfiler.

**Table 1:** Comparison of performance of the top three methodologies

Team	Core model	Competition score	Average F1	Recall at 0.7 IoU (%)	Missed at 0.7 IoU (%)	Extra at 0.7 IoU (%)
[ods.ai] topcoders	32× U-Net/FPN	0.6316	0.7120	77.62	22.38	14.55
Jacobkie	1× FC-FPN	0.6147	0.6987	69.14	30.86	15.04
Deep Retina	1× Mask-RCNN	0.6141	0.7008	68.07	31.93	10.90
CellProfiler^a^	-	0.5281	0.6280	59.35	40.65	39.55

Rows show information about each method and columns show performance metrics. Core model, type of machine-learning algorithm used to solve the task, with the number indicating how many neural networks were used in the solution. The names of neural networks are explained in the main text. Competition score, metric used during the competition to rank participants in the scoreboard (https://www.kaggle.com/c/data-science-bowl-2018#evaluation). The rest of the performance metrics were computed offline after the competition ended for analysis purposes only. Average F1 is the accuracy metric closely related to the official score, which treats the segmentation problem as a binary decision problem (correctly segmented or not) for each object. The average F1 score was computed at different IoU thresholds between target masks and estimated segmentations and then averaged across all thresholds. By setting a single IoU threshold, we could count how many objects were correctly segmented (true positives or Recall at 0.7 IoU), how many were missed (false negatives or Missed at 0.7 IoU) and how many false objects were introduced (false positives or Extra at 0.7 IoU). ^a^Note that the CellProfiler reference segmentations were generated with a different experimental protocol involving manual adjustment of pipelines for five image types in the test set. More details are provided in the Methods section.

### Best-performing solution

A. Buslaev, V. Durnov and S. Seferbekov formed the [ods.ai] topcoders team, and introduced a highly optimized, multinetwork (ensemble) model with sophisticated data augmentation and data post-processing. Coordinating all these elements in a successful solution was a major achievement because models with larger learning capacity may overfit and fail to perform well with new images. Instead, this solution generalized well to the holdout of 15 image sets. In terms of computational requirements, this was the most demanding solution, as a single image needs to be processed by 32 different neural networks using graphics processing units (GPUs). In addition, the post-processing steps need to check and combine the predicted objects from all 32 outputs. Altogether, this system was the most accurate, albeit at a high computational cost and complexity. More details in the Methods section.

### Second-best-performing solution

M. Jiang (team name: Jacobkie) presented a solution with a good balance of accuracy and speed; only a single neural network was used to process new images. Her solution introduced several innovations that can be adopted in other models, such as a loss function that penalizes errors taking into account object size (small objects have as equal weight as large objects), the use of distance maps instead of binary masks as a target for learning and pretraining with natural-image, object detection datasets. More details in the Methods section.

### Third-best-performing solution

A. Lopez-Urrutia (team name: Deep Retina) presented a solution on the basis of a single neural network that processed regions with candidate objects instead of using a fully convolutional approach. The base model is known as Mask-RCNN^33^, which is a popular architecture for object detection and instance segmentation in natural images. The simplicity of the solution was attractive, as various implementations of this solution existed and could be adapted to this problem by retraining the output layers with the right data. In addition, the Mask-RCNN model was actively investigated in the computer vision community, making innovations readily available to the nucleus segmentation problem. More details in the Methods section.

### Other solutions, participants and strategies

Apart from the top three methods, the fourth place solution^34,35^ by the team ‘Nuclear Vision’ fell 0.04 points behind third place and combined classical watershed transform with modern deep learning^36^. A stage-one U-Net (direction net) was used to predict the direction vector of a pixel inside nuclei and to the nearest nucleus boundary. Another stage-two U-Net (water transform net) estimated the watershed levels and output the masks, eroded masks and mask centers. Such methods may be useful for automatic or interactive segmentation, whereby the traditional watershed transform energy landscape is replaced by the output of learned deep networks.

The team ‘Creepy ReLU’^37^ generated synthetic images using CycleGAN^38^. They showed that color stains could be transferred from one image to another. However they did not have time to train on synthetic images; this approach might have improved the top solutions in the competition.

On a sociological note, the competition brought experts from different domains together to share software and cloud resources and to develop ideas. In particular, team ‘minerva.ml’^39^ open sourced their code and development process at the start of the competition and provided a cloud-based platform that Kagglers could use. The author of the open source Matterport Mask-RCNN implementation (https://github.com/matterport/Mask_RCNN) also participated in the competition^40^ and provided a complete software pipeline tool for training, visualization and submission. We noted that the third and fifth-place teams in the competition based their solutions on this Matterport implementation. This shows that the quality of open source implementation was high, included suitable options and parameters and could be used off the shelf.

## Discussion

The 2018 Data Science Bowl presented the challenge of automatically finding nuclei in a large variety of unseen microscopy images, with no configuration step. This was the first documented attempt to produce a model that could segment the stained nuclei of cells in 15 biological experiments, across experimental conditions, acquisition equipment and source laboratory. The main goal of the challenge was to investigate generic segmentation strategies that could be automatically applied to many imaging experiments with no further user intervention. This approach may reduce the time to quantify images, empowering future generations of biologists to adopt and run more quantitative imaging experiments for research and clinical practice.

Training automated nucleus segmentation tools using modern machine-learning approaches requires collecting annotated examples. The 2018 Data Science Bowl created a resource of diverse images contributed by numerous biological laboratories and manually annotated by a team of expert biologists at the Broad Institute. All those data are now publicly available with public domain licenses to facilitate future scientific research as well as industrial development. We hope others in the wider bioimaging community will contribute more images and annotations to grow this resource with additional experimental variations, including unstained brightfield and electron microscopy, as well as many other common image modalities that were not included in our study.

The challenge attracted participation from different teams in the data science community, who made all types of contributions, learned together and collaborated to understand the problem better and make progress toward the proposed goal. Solutions presented by several participants achieved the goal of a single model able to segment various microscopy images with no intervention. The experimental results indicate that nucleus segmentation could be fully automated, requiring no manual settings or image processing expertise from users, while still providing improved accuracy versus the evaluated tools. Higher accuracy may be possible through a larger, more diverse training set and by incorporating the latest advances in machine learning and computer vision research.

The top participants presented solutions on the basis of fully convolutional networks (U-Nets and FPNs) or Mask-RCNN. These two approaches were widespread during the competition; what distinguishes the winners was a combination of pre-processing and post-processing techniques, as well as the application of best practices during training (mostly data balancing and data augmentation). A common theme among the top competitors was the use of data augmentation during training and testing, including color shifts to make networks color invariant, and scaling methods to address object size challenges. Interestingly, all top three solutions used a ranking strategy to select the best segmentation masks from several candidates predicted by the base models. While this is common practice for RCNN-like models (third-best solution), the top two models also created their own strategies to achieve a similar effect with fully convolutional networks.

The results present a successful proof of concept that deep learning is indeed capable of delivering accurate results without user interaction. However, even though the top models are publicly available, they still require computational expertise to be applied to images. A user-friendly tool is needed to bridge the gap between these cutting-edge solutions and everyday biomedical practice, similarly to what the NucleiAIzer system proposes^21^. We also found that data availability is a limitation to reach top performance for various image types, thus, additional efforts are needed to collect and annotate more data to expand the applicability of future systems. The generalization ability of models may also be evaluated in other datasets not used during the Data Science Bowl challenge, such as the Cell Tracking Challenge and others. Other aspects of usability remain to be addressed. For instance, if there are mistakes in the segmentation, how can these models efficiently and easily take feedback from humans to correct segmentation errors? The results of the 2018 Data Science Bowl are a first step toward creating a generic system for segmenting the nucleus of cells in every microscopy image. Future work could expand the dataset to cover missing major microscopy imaging types, such as unstained brightfield images and three-dimensional images. Following the strategy laid out here, models could also be constructed to segment cell structures in addition to the nucleus, such as cell borders and organelles.

## Methods

### Dataset

The image sets were donated by multiple laboratories studying different aspects of cell biology. The names and credits are listed in Supplementary File 1. A total of 841 images were collected, representing a wide variety of nuclei observed under different experimental conditions and imaged with various staining protocols. Our goal was to collect as many independent biological experiments as possible to create a resource that contains enough technical and biological variability to train generic nucleus segmentation models.

In total, the dataset contained images from more than 30 different biological experiments, which were split into 16 experiments for training (670 images) and first-stage evaluation (65 images) and exactly 15 experiments for the second-stage evaluation (106 images). The number of experiments represented in the training and first-stage evaluation is approximate because these include images from public or anonymous sources without metadata to confirm the exact number. Holding 15 experiments for the second-stage evaluation allowed us to simulate the realistic evaluation case of bringing newly acquired images for segmenting their nuclei. See Supplementary Note 1 for more statistics and details about the dataset.

#### Annotation strategy

Overall, the image set was annotated with 29,464 individual nuclei in the training set, 4,152 in the first-stage test set and 3,717 in the second-stage test set, for a total of 37,333. The annotations were created by expert biologists who manually delineated each object in the images using one of two tools: (1) an assisted annotation tool that precomputed superpixel segmentations to facilitate the selection of regions in the foreground or background; and (2) the GIMP image editing software to create annotation masks by coloring individual pixels outlining each nucleus.

The assisted annotation tool made an initial over-segmentation of the image using the simple linear iterative clustering superpixels algorithm^41^. The annotators could then color each superpixel with one of four colors to indicate what regions correspond to objects and what others to background. Objects were required to have different colors if they were touching each other. Superpixels are very helpful to reduce the amount of annotation time, but also may contain systematic noise because their boundaries are not necessarily perfectly aligned with the real object boundary. This strategy was used for training images only to facilitate large scale annotation; for test images, we used the per-pixel annotation strategy using GIMP to score participants with respect to masks drawn 100% manually. Human annotations were not post-processed to avoid introducing unintended artifacts. See Supplementary Note 5 for more details.

#### Image modalities

In this dataset, we included 2D light microscopy images of stained nuclei. The majority of the images in this dataset came from fluorescent images with cells of different sizes and various types, primarily stained with DAPI or Hoechst. The dataset also included tissue samples stained with hematoxylin and eosin, displaying structures from a diversity of organs and animal models. The image collection was organized to include different technical settings and a variety of biologically different experiments. We excluded phase-contrast, differential interference contrast and other image modalities because during the data collection period we did not find image sets or donating laboratories that could make these images available in the public domain.

### Evaluation

#### Performance metrics

The evaluation strategy was on the basis of identifying object-level errors. This was accomplished by matching target object masks with predicted objects submitted by participants and then computing true positives and false positives. In order to match target masks and predicted objects, the intersection-over-union (IoU) score was computed for all pairs of objects using ${\mathrm {IoU}} = \frac{|{{\mathrm {A}} \cap {\mathrm {B}}}|}{|{{\mathrm {A}} \cup {\mathrm {B}}}|}$, where A and B are two objects, and the operator | | measures area.

A minimum IoU threshold *t* was selected to identify correctly segmented objects and any other predicted segmentation mask below the threshold was considered an error. With all true positives (TP), false positives (FP), true negatives (TN) and false negatives (FN), we created a confusion matrix and computed precision (*P*), recall (*R*) and F1 scores, using a fixed IoU threshold *t* as follows:

$$\begin{array}{*{20}{l}}{\mathrm{P}}\left( t \right) &=& \frac{{{\mathrm {TP}}\left( t \right)}}{{{\mathrm {TP}}\left( t \right) + {\mathrm {FP}}\left( t \right)}}\\{\mathrm {R}}\left( t \right) &=& \frac{{{\mathrm {TP}}\left( t \right)}}{{{\mathrm {TP}}\left( t \right) + {\mathrm {FN}}\left( t \right)}}\\{\mathrm {F1}}\left( t \right) &=& \frac{{{\mathrm {2TP}}\left( t \right)}}{{{\mathrm {2TP}}\left( t \right) + {\mathrm {FP}}\left( t \right) + {\mathrm {FN}}\left( t \right)}}\end{array}$$

We used increasing IoU thresholds to estimate shape-matching accuracy. When a segmentation covered the target mask perfectly, the IoU score was 1 and the object was correctly detected no matter which threshold was used. In practice, segmentations can only approximate the real shape of the object, so at certain coverage threshold the object was missed. This test estimated how well segmentations matched the shape of manually defined target masks. Then, the official competition score *S*, was defined in terms of type I and II errors, using multiple IoU thresholds as follows:

$$S = \frac{1}{{\left| T \right|}}\mathop {\sum}\nolimits_{t \in T} {\frac{{{\mathrm {TP}}\left( t \right)}}{{{\mathrm {TP}}\left( t \right) + {\mathrm {FP}}\left( t \right) + {\mathrm {FN}}\left( t \right)}}}\, ,{\mathrm{where}} \,T = \left\{ {0.10,0.15,...,0.95} \right\}$$

For some of the results reported in this paper, we also computed F1, precision and recall in an aggregated manner, similar to the official score. The competition evaluation score is described in detail in Supplementary Note 2.

#### Two-stage evaluation protocol

The data challenge was on the basis of a two-stage evaluation protocol with one training set and two holdout sets. The training and first-stage holdout sets were available to competitors during a period of 2.5 months for calibrating the algorithms. The first-stage holdout target masks were not directly accessible to the competitors but instead used to test their submitted segmented images against, yielding a numerical score. Participants were allowed to make a maximum of five submissions per day to obtain feedback about their performance, and they could select two submissions for evaluation and ranking. Depending on the scores obtained from these submissions, they decided to tune their methods and submit again later during the competition.

The second-stage holdout set was released during the final period of the competition, giving participants only 1 week to process 3,200 images. These images had an estimated 100,000 single nuclei, only 106 (3%) images had manually defined target masks useful for scoring, and the competitors did not know which images were going to be evaluated. This approach was enforced to prevent competitors from using extremely slow solutions, hand-outlining results or choosing among a large number of algorithms or settings by visually verifying the results. The same submission rules applied during the second-stage evaluation, allowing participants to submit at most five times per day and select only two submissions for final scoring.

The segmented images produced by the top solutions were manually screened and presented naturally occurring errors produced by automated solutions; none of them had signs of hacking or cheating. This experimental procedure was as rigorous as those used in other initiatives organized by scientists for other scientists. Kaggle has a long history of experience working with scientists to define these experimental settings and has a good pool of best practices for data science that were enforced to assure the validity of the results and identify hacking.

#### Grouping of images

The guiding principles for creating the five groups of images in the dataset was visual similarity on the basis of image colors and object sizes. The goal was to facilitate the application of classical image segmentation algorithms, which most heavily relied on (1) the nuclei and background colors (white versus black, purple versus white, purple versus pink, back versus gray); and (2) the approximate size of nuclei in the image. The number of groups constructed was kept as low as possible while maintaining the ability to create a robust analysis workflow for each group.

This organization in five groups may reflect staining protocols and microscopy techniques used in the experiments. However, that information was not explicitly used for determining the assignment of images to groups, it was all on the basis of visual inspection. In fact, the analyst that created the groups and designed classical segmentation workflows had no knowledge of experimental details of the test sets. This was intentional, with the purpose of making generic segmentation solutions with minimal assumptions, similarly to the conditions presented to participants of the competition.

The five groups of images were also used to conduct data analysis of segmentation performance of competitors. Most of the results presented in this paper have been organized around these five generic groups of images. However, this information was not provided to participants of the competition, for them, the entire dataset contained varied example microscopy images with a single object of interest, the nucleus.

#### Reference segmentations

Our goal was to evaluate the contribution of nucleus segmentation methods proposed by participants of the challenge, considering that these methods worked across a variety of experiments with no user intervention. The most appropriate baseline was an existing strategy that could be applied to any image (within the constraints laid out, 2D images stained for nuclei) and produce accurate results with no human interaction. Given that there was no such method in existence, we approximated it by taking an approach that used as little human time as possible. As an approximation for quantitative reference, we do not call the approach baseline segmentations, but rather reference segmentations.

We chose CellProfiler v.3.1.5 as the tool to create reference segmentations given its flexibility to configure robust pipelines on the basis of well-established algorithms, while investing a minimum amount of time. CellProfiler is a powerful open source tool for microscopy image analysis that includes a variety of fundamental image processing algorithms in a modular way. The algorithms can be organized in a computational graph (pipeline) that takes images as inputs and produces various types of outputs. Importantly, a pipeline is defined using a user-friendly interface and can run complex operations without the need to write a single line of code. These properties made CellProfiler a good reference for models in the competition, because the goal of the challenge was to investigate generic nucleus segmentation methods with minimal user interaction.

Five custom pipelines were designed, one for each of the image types in the test set, with the goal of evaluating the classical algorithms implemented in CellProfiler. A single classical segmentation pipeline was unlikely to work well in the variety of images represented in the test set, thus, we adapted the best practices reported in the literature for each group of images. This approach had an advantage over the deep-learning models tested during the competition because the image sets were manually organized and processed with special routines according to their type. Deep-learning models were not expected to have any manual intervention from users.

The pipelines were designed with three major sequential steps: (1) pre-processing to transform the image into a grayscale matrix, where nuclei were observed as relatively smooth white shapes on a black background; (2) segmentation of the grayscale image using thresholding, distance transforms and watershed on the basis of approximate expected nuclear size; and (3) segmentation revision using seeded watershed on the basis of the previous segmentation and additional nuclear size priors. Steps 2 and 3 were on the basis of the Identify Primary Objects and Identify Secondary Objects modules of CellProfiler, which are documented in the cell segmentation literature^5,8,42–46^. Pipelines are available online at https://github.com/carpenterlab/2019_caicedo_submitted/tree/master/pipelines, with annotations in each module to describe that module’s function in the overall pipeline. Supplementary Figs. 1 and 2 illustrate the corresponding computational graphs for each of the five pipelines designed.

Importantly, we did not optimize the algorithm parameters for each experiment, but rather tuned the methods to be as generic and automatic as possible for each group. In that sense, the solution may be suboptimal, but is representative of the daily use of image analysis. Perhaps some errors can be fixed by tweaking parameters for individual images or experiments, but we are not interested in these types of solutions, given that our goal is to minimize manual overhead work.

### Top three solutions

#### Best-performing solution

The system was on the basis of an ensemble strategy with eight fully convolutional neural network architectures; for each, four replicate models were trained resulting in a total of 32 trained segmentation networks in the final solution. All eight base architectures followed the encoder–decoder principle to process an input image and generate the segmentation map in the output. Six of these base architectures used U-Net-like decoders^17^ and the other two used a FPN^47^ decoding scheme. The encoders included Resnets (34, 50, 101, 152)^48,49^, Dual Path Networks^50^ and Inception-Resnet^51^.

The team reported that properly modeling the target masks for training U-Net or FPN models was critical to achieve the best performance. In their final solution, they incorporated an approach on the basis of nuclei masks separated by artificially generated boundaries. Then, the task of a segmentation network was to classify pixels into three types: background pixels, interior of cells and boundary pixels. The best performance was obtained when the boundary pixels were marked between only touching cells. Previous works have also considered modeling the target masks in a similar way^18,20,52^, which is equivalent to a semantic segmentation approach to separate instances.

The combination of outputs from the 32 networks was performed in three steps: first, aggregation of predicted masks using the mean of all, second using a ranking model to filter out noisy predictions and third applying a watershed algorithm to refine boundaries. The ranking model of the second step used classical morphological features extracted from each candidate nucleus. These features were used to train a regression model (gradient-boosted trees) that learned to predict IoU scores from ground-truth examples. During test, each candidate object was post-processed in this way to estimate how well it aligned with a potentially real object. This strategy allowed scoring many segmentation masks and ranking them from the most to the least promising one, which was useful to identify and remove false predictions.

The team focused on preventing overfitting with two strategies: (1) using neural networks pretrained on the popular ImageNet database^53^ as feature encoders for all eight architectures; and (2) using heavy data augmentation to harness the training examples as efficiently as possible. A total of 24 augmentation routines, including channel shuffling, color inversion and object copying, were used for training all models. Additional microscopy images from publicly available databases were also employed by this team to expand the pool of training examples, including Wikimedia images, which were manually annotated by them. The open source code is available at https://github.com/selimsef/dsb2018_topcoders

#### Second-best-performing solution

The system was a single neural network model on the basis of the FPN architecture^47^. The solution introduced two customized output layers, each producing multichannel-relative position masks with estimated distances of each nucleus to their boundaries in four directions (vertical, horizontal, 45 degrees and 135 degrees). Relative position masks are analogous to the ‘deltas’ or distances of pixels with respect to anchor reference points in region proposal networks^54^. Importantly, the coordinate maps were computed densely for every pixel in the interior of nuclei, whereas pixels in the background were set to zero. Also, relative position masks were post-processed and transformed into boundaries, refined with watershed and ranked by consistency between local and global scores to select the final set of nonoverlapping masks.

The backbone FPN in this solution was pretrained on the ImageNet^53^ and COCO^55^ datasets using the Matterport implementation of the Mask-RCNN^33^ framework. The two output layers were trained using a multitask framework. A new loss function was introduced to penalize instance errors by the size of objects, balancing the contribution of errors by small objects with respect to large objects. Various data augmentation techniques were applied during training and testing and no external data were used. Test-time data augmentation consisted of making predictions on transformed versions of the test image (such as scaling or rotation) and then integrating those predictions in a single output. The open source code is available at https://github.com/jacobkie/2018DSB.

#### Third-best-performing solution

This solution was a Mask-RCNN model, pretrained with the COCO dataset^55^ for detecting and segmenting objects in natural images. The solution included data augmentations that were meaningful in the biological context, including simulated magnifications of microscopes by scaling images up and down artificially. Aspect ratio modifications, flips and rotations were also used. The training dataset was balanced with respect to types of image, although no special analysis was used to determine the image type; only image size was considered to oversample underrepresented images with random augmentations. Additional data augmentations were also applied during training, and the model was not retrained for the stage-two evaluation with additional data or more iterations. This leaves room to investigate the role of more data when using this model.

To generate segmentations for new images, the participant introduced 15 test-time data augmentations, which looked at the test image under different transformations and aggregated the predictions in a single output. These transformations included rotations in different angles, image scaling and color shifts. This was one of the differences of the participant’s approach with respect to others that also used Mask-RCNN without the same success. The participant also reported that the simpler post-processing techniques, such as morphological dilation, may reach similar performance and that the parameter configuration and data augmentation during training seemed to be more important according to his experiments. The open source code is available at https://github.com/Lopezurrutia/DSB_2018.

### Reporting Summary

Further information on research design is available in the Nature Research Reporting Summary linked to this article.

## Online content

Any methods, additional references, Nature Research reporting summaries, source data, statements of code and data availability and associated accession codes are available at 10.1038/s41592-019-0612-7.

## Supplementary Information

### Integrated supplementary information

Supplementary Figure 1 Computational graph of the pipelines used for three groups of images: grayscale tissue, purple tissue, and pink and purple tissue. All pipelines finish with the Identify Primary Objects (IPO) module, which runs thresholding, distance transforms and watershed on the grayscale image produced in the immediately previous step. The modules before IPO aim to transform the input image into a grayscale matrix suitable for segmentation.

Supplementary Figure 2 Computational graph of the pipelines used for the two fluorescent groups of images. Both pipelines make use of the Identify Primary Objects (IPO) module as well as the Identify Secondary Objects (ISO) module. Both modules are based on thresholding and watershed using seeds computed from distance transforms and previously identified objects. These were needed due to the large variability of experiments and nucleus phenotypes present in the data. Other modules aim to reduce and filter noise to prepare the image for segmentation.

Supplementary Figure 3 Estimation of inter-observer variability. a) Each small point in the plot corresponds to one object. The y axis reports the intersection-over-union (IoU) score between compared objects and the x axis reports the pairs of subjects or methods being compared. Large points are the median of all object scores. The color of small points corresponds to the type of image the object comes from (legend in the bottom-right). Nuclei from grayscale tissue images is harder to segment for computational methods, while human annotators generally agree on their masks. Purple points display disagreement more often, regardless of the pairs being compared. b) pairs being compared with measurements of agreement in overlap (IoU) and accuracy (F1 score @ 0.7 IoU). Human annotators (green row) reach high object overlap agreement, but the top model (blue rows) agrees more often with both humans than what they agree between themselves. However, the model has slightly more disagreement with humans in terms of accuracy, which means the model misses a few objects more frequently than humans do. The CellProfiler reference displays substantial disagreement with humans in terms of overlap and accuracy.

Supplementary Figure 4 Differences in image annotations in 5 selected image examples. Top row: original images. Next rows: comparison of segmentation between two subjects or methods, one in blue and the other in red. Blue and red objects or outlines indicate annotations introduced by the corresponding observer that do not match the objects or outlines made by the other observer. Ideally, the map should be completely white, which would mean that all annotations are perfectly aligned.

Supplementary Figure 5 Differences in boundaries between annotators and models. Human annotations introduce subjective noise in the boundaries. The top model learned to produce smooth curves that are close to the edge of the object. This example illustrates how the top model’s boundaries can agree more often with both annotators, while humans may disagree on small boundary details. The CellProfiler segmentations, produced with the Watershed algorithm, fit low-level intensity signal that is not as smooth.

Supplementary Figure 6 Comparison of error rates of the three best performing solutions along with the CellProfiler reference segmentations. The black bars and red dots show global performance for each participant, while color bars indicate performance for each of the visually distinctive groups in the image collection.

Supplementary Figure 7 Distribution of precision, recall and F1 scores obtained by all the 739 participants in the second stage evaluation, discriminated by image type. Points in the plot correspond to the top three participants and the CellProfiler segmentation reference. All metrics are measured at the 0.7 intersection over union threshold, which can be interpreted as the requirement that objects have to overlap symmetrically at least with 70% of their area, to be counted as a true positive.

### Supplementary information

Supplementary Information Supplementary Figs. 1–7, Data Science Bowl Strategy Document and Supplementary Material.

Reporting Summary

Supplementary Notes Supplementary Notes 1–7.

Supplementary Tables Supplementary Tables 1–3.

## Data Availability

The methods of the top three participants are publicly available with usage instructions and parameters described by their creators. The authors of this manuscript did not implement and do not maintain these repositories. All the credits and copyright belong to the top three participants who created these models for the 2018 Data Science Bowl challenge. We analyzed data from the participants, downloaded from the Kaggle website using administrative permissions provided by them. Custom code was developed to investigate the patterns and trends in the submitted entries. All the data used to complete the analysis are not made publicly available owing to Kaggle’s privacy policies. Instead, aggregated results and code are available at https://github.com/carpenterlab/2019_caicedo_dsb.

## Data availability

The dataset is publicly accessible in the Broad Bioimage Benchmark Collection with accession number BBBC038.
