# Publisher Correction: Nucleus segmentation across imaging experiments: the 2018 Data Science Bowl

**DOI:** 10.1038/s41592-020-0733-z

**Published:** 2020-01-22

**Authors:** Juan C. Caicedo, Allen Goodman, Kyle W. Karhohs, Beth A. Cimini, Jeanelle Ackerman, Marzieh Haghighi, CherKeng Heng, Tim Becker, Minh Doan, Claire McQuin, Mohammad Rohban, Shantanu Singh, Anne E. Carpenter

**Affiliations:** Broad Institute of MIT and Harvard, Cambridge, MA USA

**Keywords:** Machine learning, Image processing

Correction to: *Nature Methods* 10.1038/s41592-019-0612-7, published online 21 October 2019.

This paper was originally published under standard Springer Nature copyright (© The Author(s), under exclusive licence to Springer Nature America, Inc.). As of the date of this correction, the paper is available online as an open-access paper under a Creative Commons Attribution 4.0 license.

