# Supplementary figures and images for: Nucleus segmentation across imaging experiments: the 2018 Data Science Bowl
Source: Nat Methods. 2019 Oct 21;16(12):1247–53. doi: 10.1038/s41592-019-0612-7 (PMC6919559; doi:10.1038/s41592-019-0612-7)

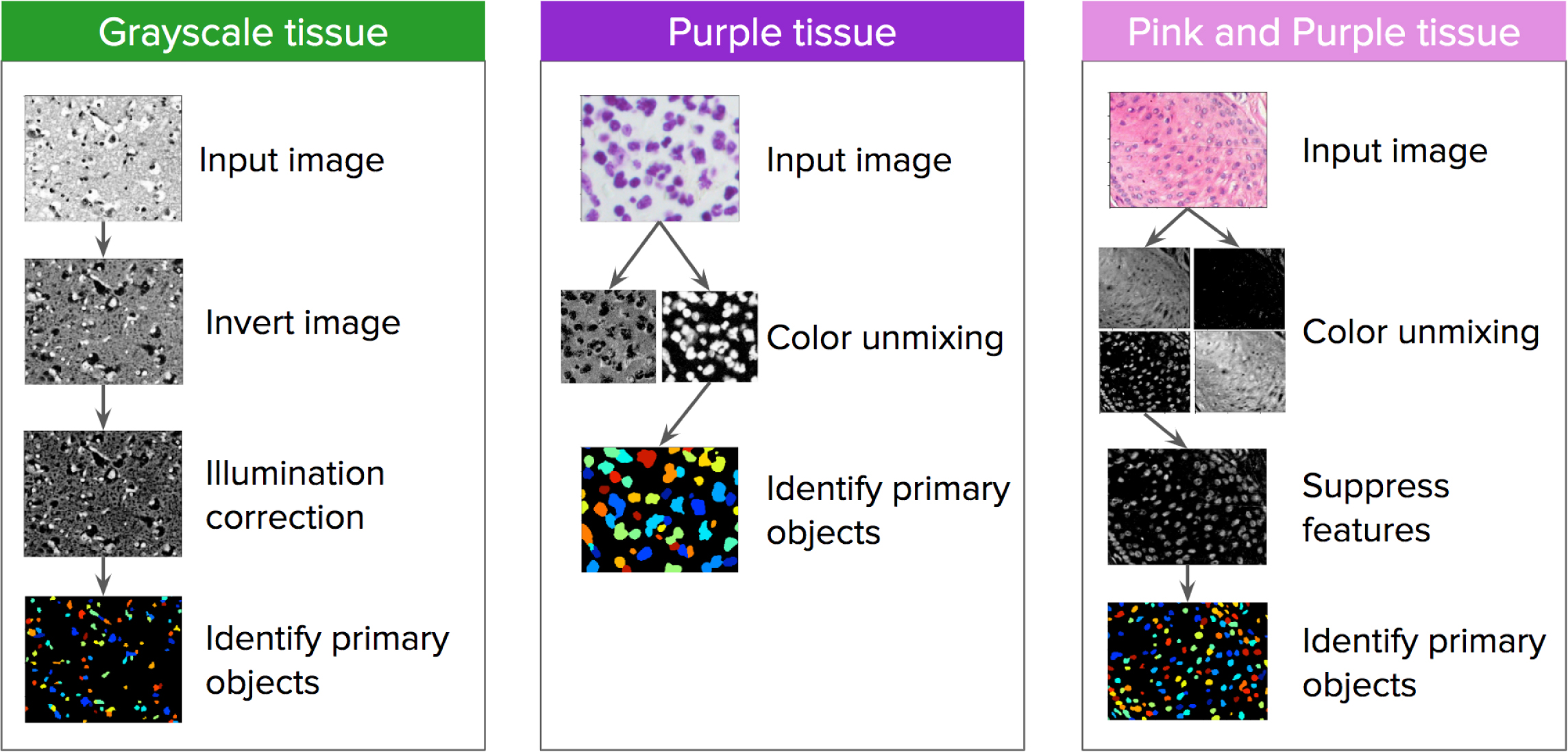

Supplement: Computational graph of the pipelines used for three groups of images: grayscale tissue, purple tissue, and pink and purple tissue. — All pipelines finish with the Identify Primary Objects (IPO) module, which runs thresholding, distance transforms and watershed on the grayscale image produced in the immediately previous step. The modules before IPO aim to transform the input image into a grayscale matrix suitable for segmentation. [file 41592_2019_612_Fig4_ESM.jpg]

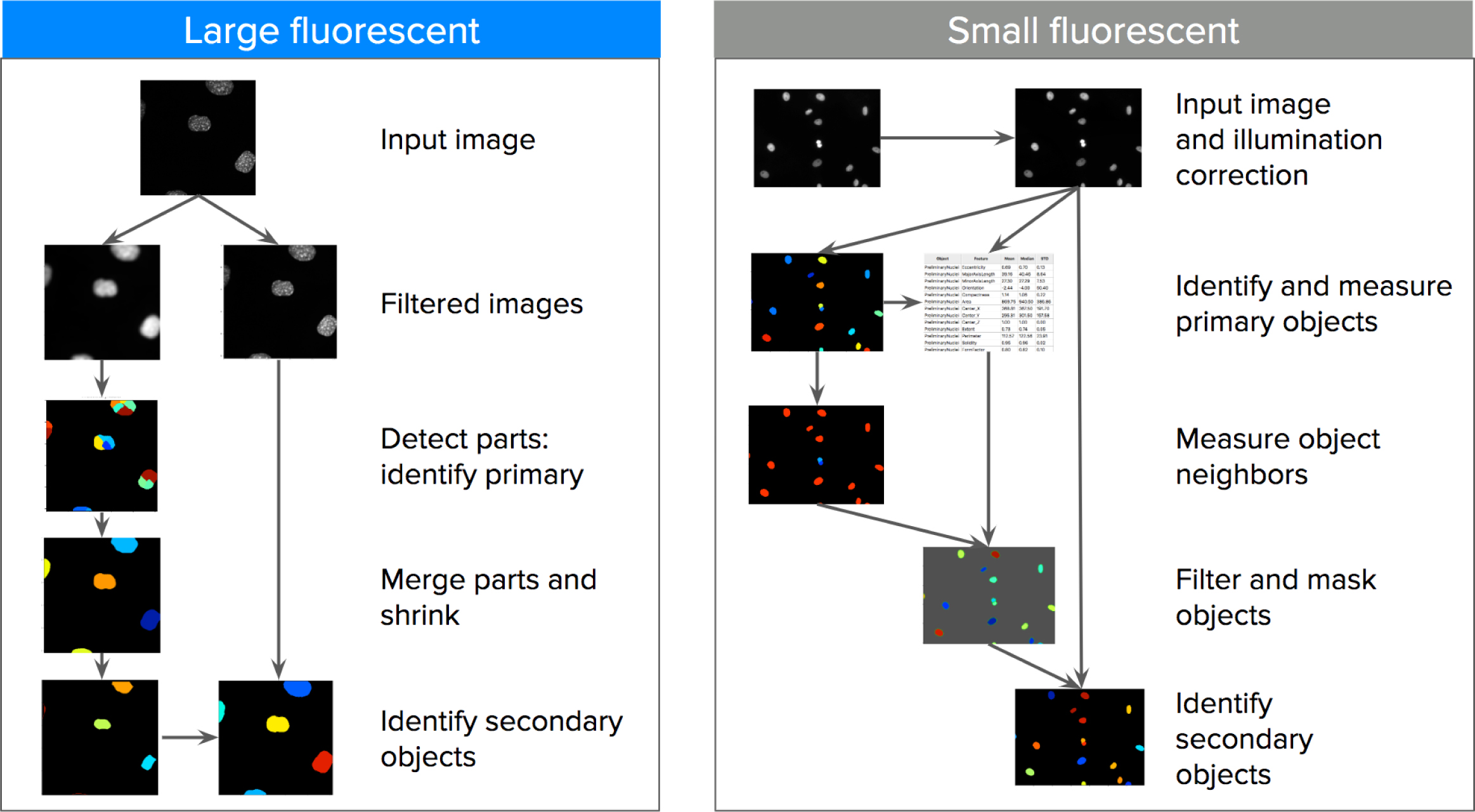

Supplement: Computational graph of the pipelines used for the two fluorescent groups of images. — Both pipelines make use of the Identify Primary Objects (IPO) module as well as the Identify Secondary Objects (ISO) module. Both modules are based on thresholding and watershed using seeds computed from distance transforms and previously identified objects. These were needed due to the large variability of experiments and nucleus phenotypes present in the data. Other modules aim to reduce and filter noise to prepare the image for segmentation. [file 41592_2019_612_Fig5_ESM.jpg]

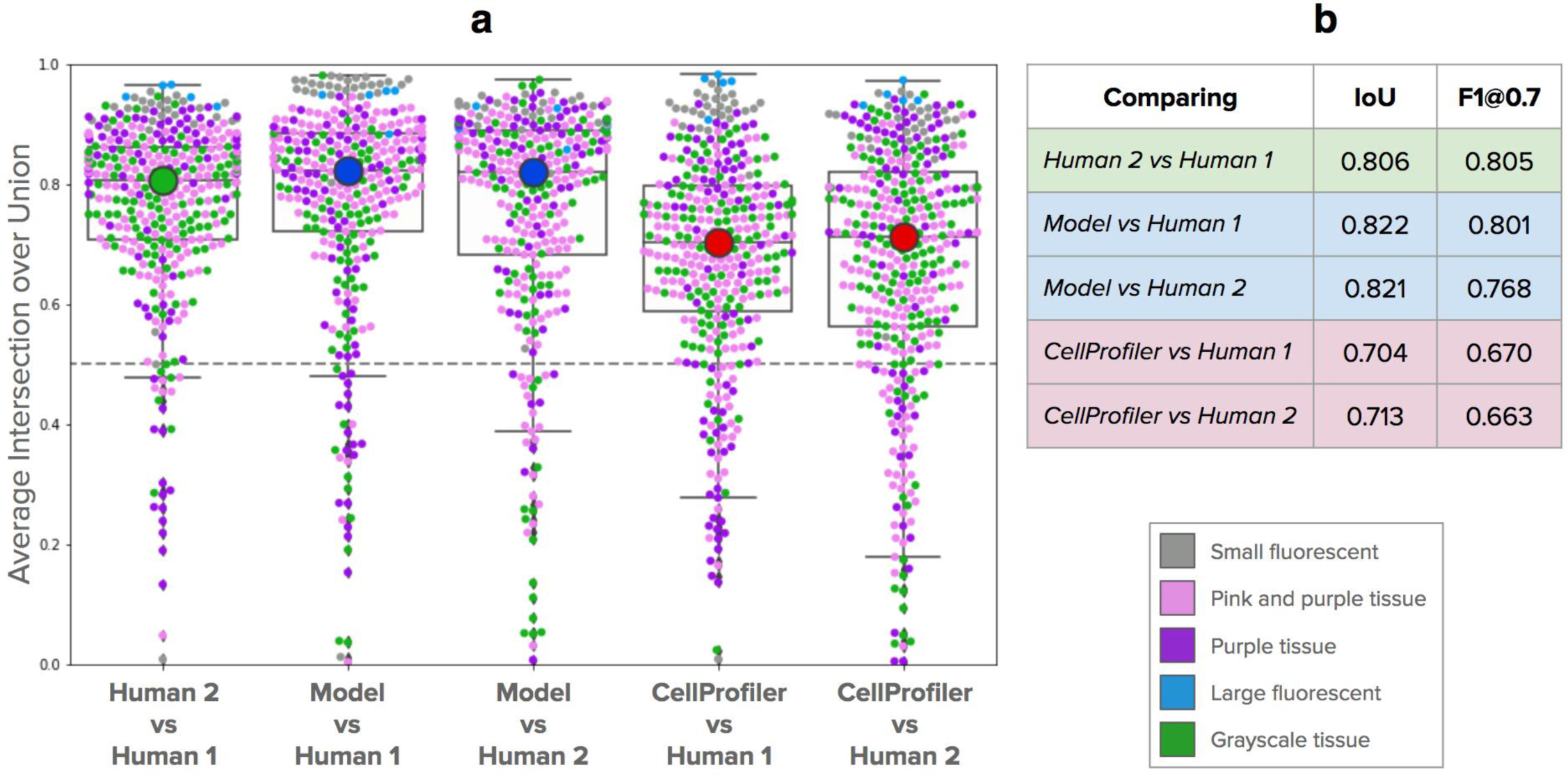

Supplement: Estimation of inter-observer variability. — a) Each small point in the plot corresponds to one object. The y axis reports the intersection-over-union (IoU) score between compared objects and the x axis reports the pairs of subjects or methods being compared. Large points are the median of all object scores. The color of small points corresponds to the type of image the object comes from (legend in the bottom-right). Nuclei from grayscale tissue images is harder to segment for computational methods, while human annotators generally agree on their masks. Purple points display disagreement more often, regardless of the pairs being compared. b) pairs being compared with measurements of agreement in overlap (IoU) and accuracy (F1 score @ 0.7 IoU). Human annotators (green row) reach high object overlap agreement, but the top model (blue rows) agrees more often with both humans than what they agree between themselves. However, the model has slightly more disagreement with humans in terms of accuracy, which means the model misses a few objects more frequently than humans do. The CellProfiler reference displays substantial disagreement with humans in terms of overlap and accuracy. [file 41592_2019_612_Fig6_ESM.jpg]

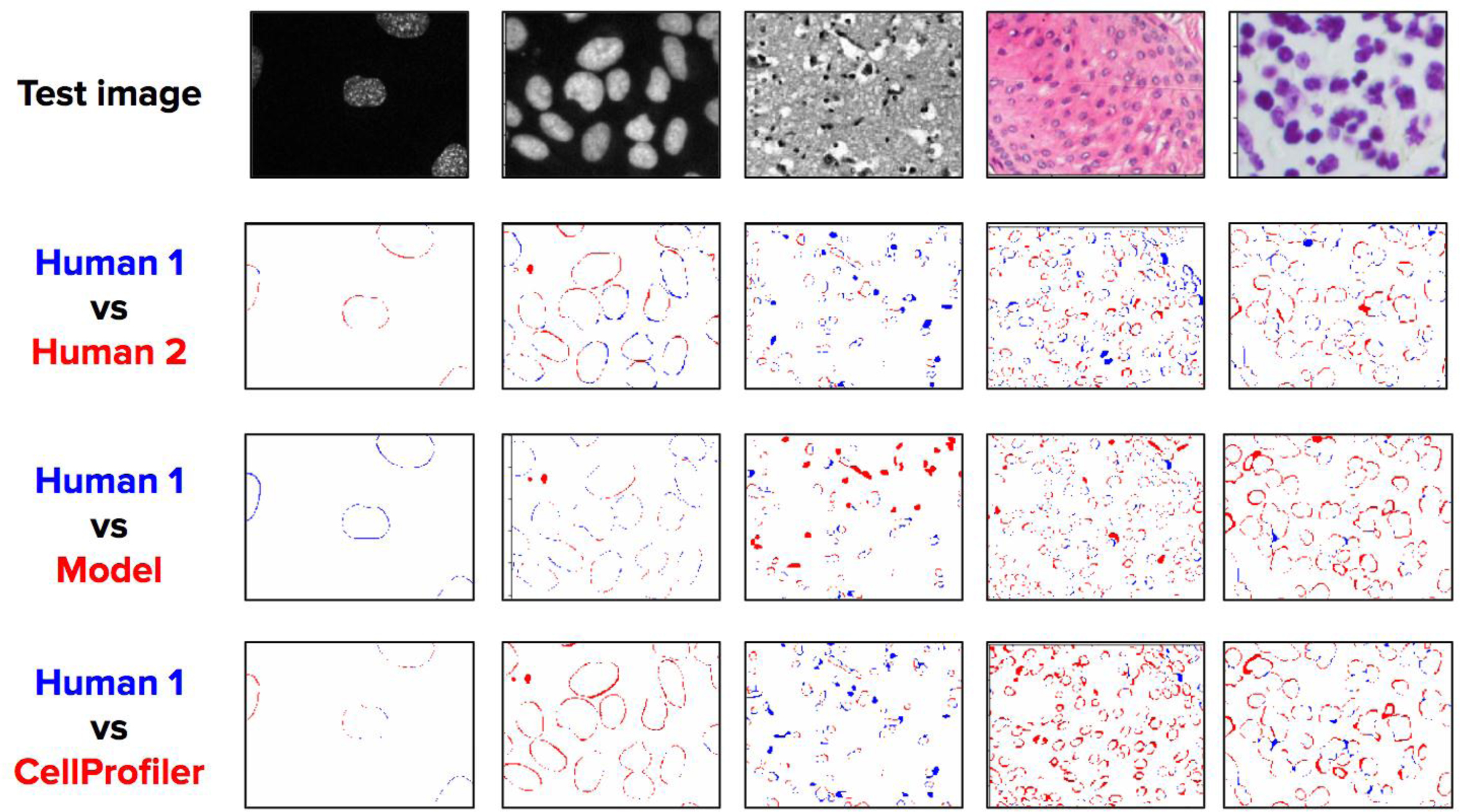

Supplement: Differences in image annotations in 5 selected image examples. — Top row: original images. Next rows: comparison of segmentation between two subjects or methods, one in blue and the other in red. Blue and red objects or outlines indicate annotations introduced by the corresponding observer that do not match the objects or outlines made by the other observer. Ideally, the map should be completely white, which would mean that all annotations are perfectly aligned. [file 41592_2019_612_Fig7_ESM.jpg]

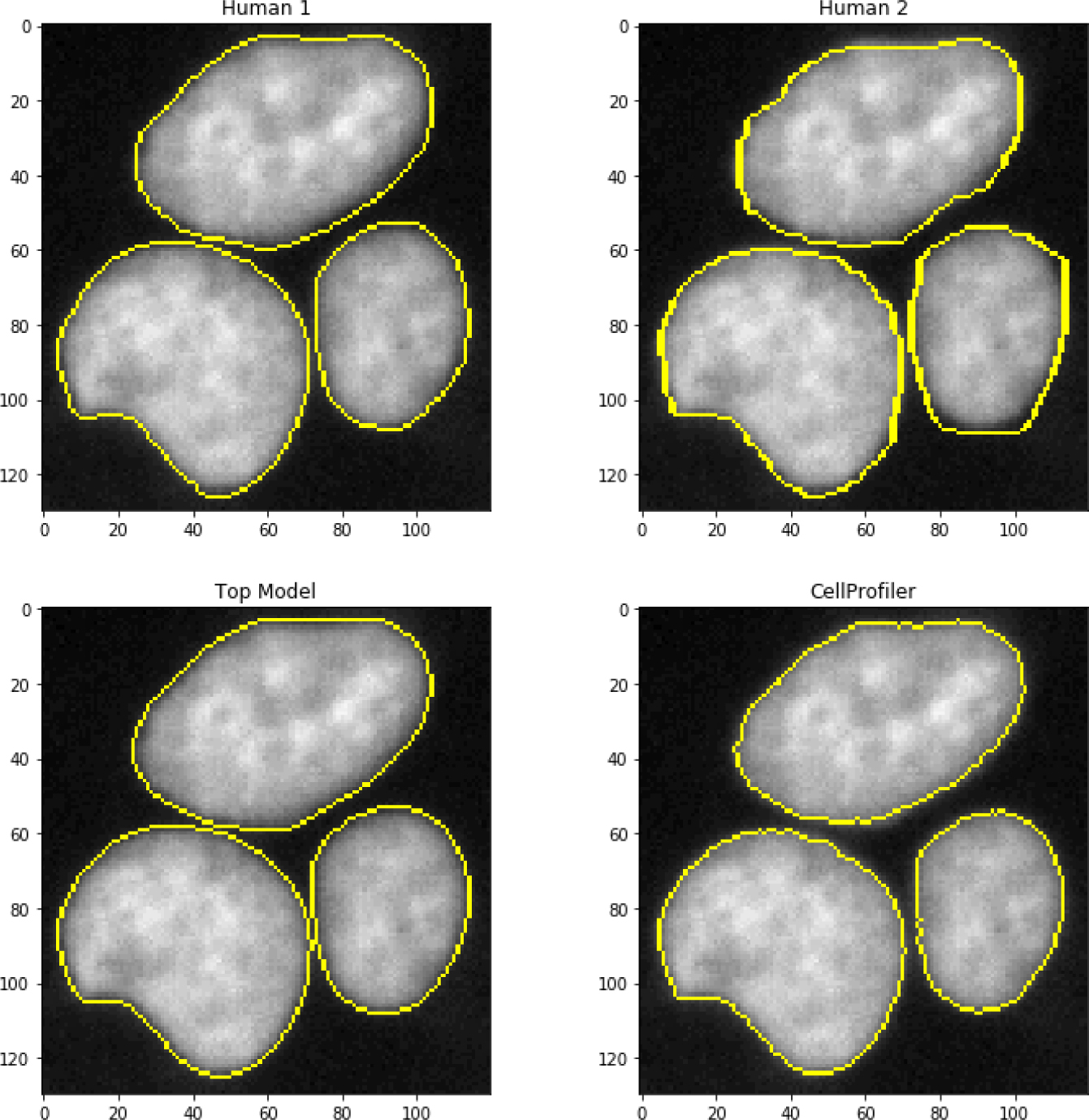

Supplement: Differences in boundaries between annotators and models. — Human annotations introduce subjective noise in the boundaries. The top model learned to produce smooth curves that are close to the edge of the object. This example illustrates how the top model’s boundaries can agree more often with both annotators, while humans may disagree on small boundary details. The CellProfiler segmentations, produced with the Watershed algorithm, fit low-level intensity signal that is not as smooth. [file 41592_2019_612_Fig8_ESM.jpg]

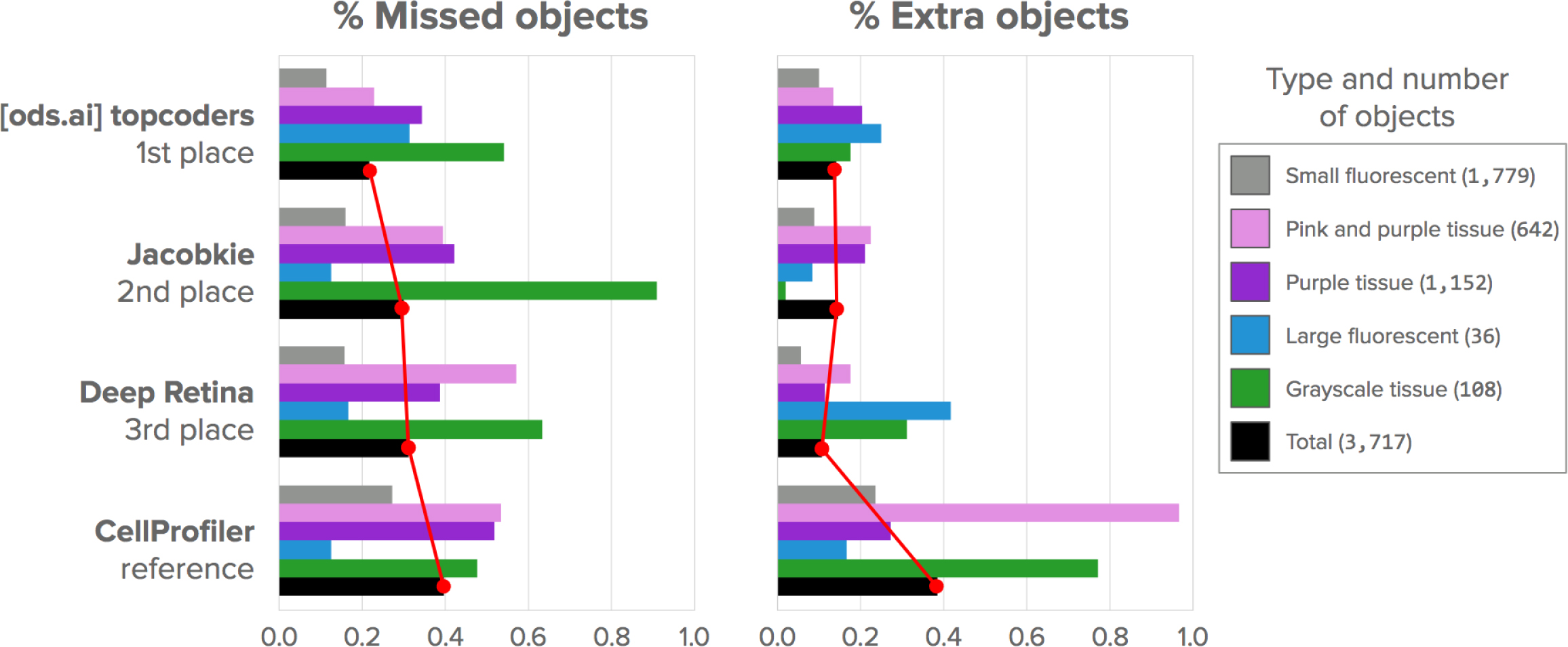

Supplement: Comparison of error rates of the three best performing solutions along with the CellProfiler reference segmentations. — The black bars and red dots show global performance for each participant, while color bars indicate performance for each of the visually distinctive groups in the image collection. [file 41592_2019_612_Fig9_ESM.jpg]

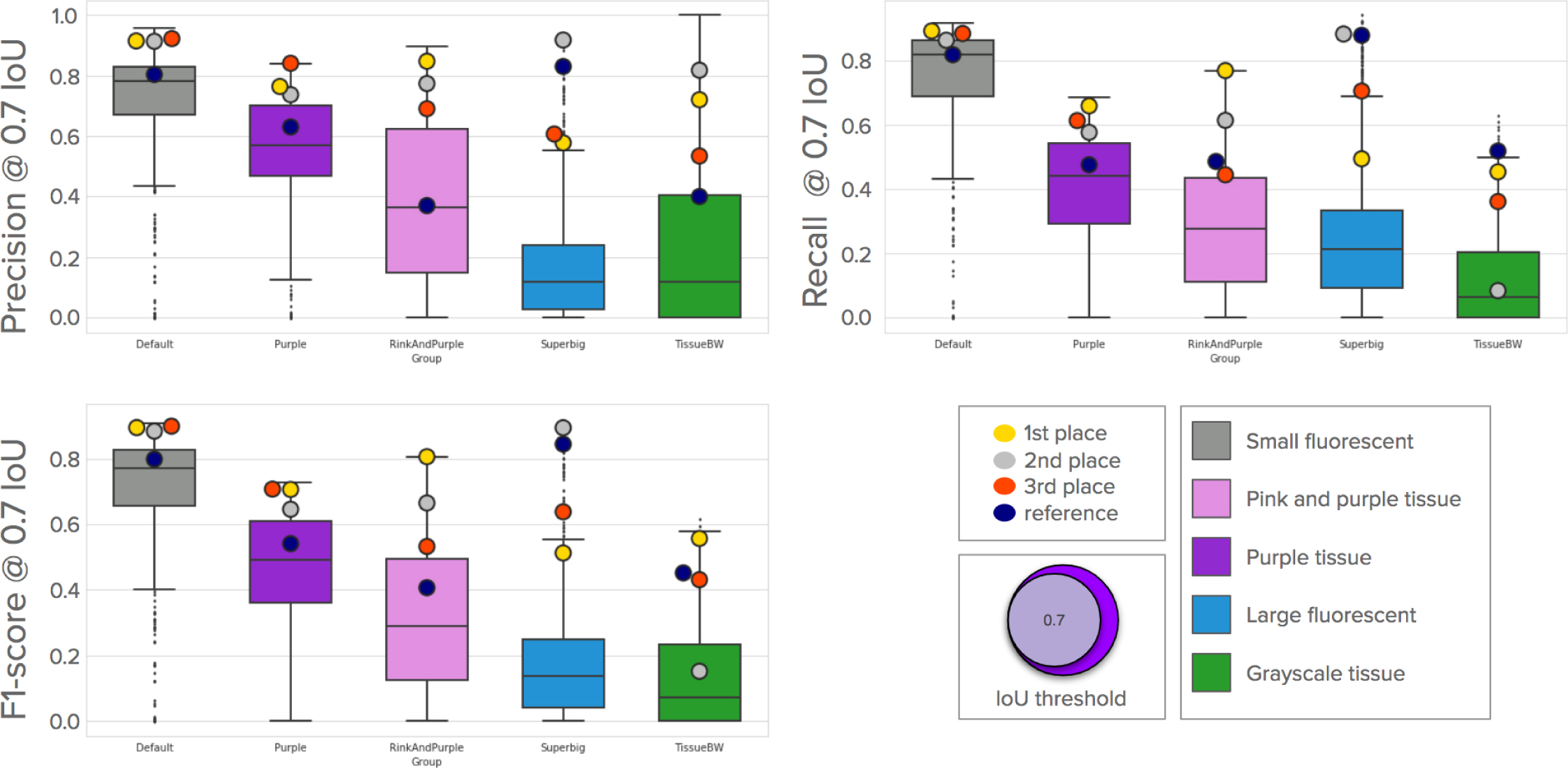

Supplement: Distribution of precision, recall and F1 scores obtained by all the 739 participants in the second stage evaluation, discriminated by image type. — Points in the plot correspond to the top three participants and the CellProfiler segmentation reference. All metrics are measured at the 0.7 intersection over union threshold, which can be interpreted as the requirement that objects have to overlap symmetrically at least with 70% of their area, to be counted as a true positive. [file 41592_2019_612_Fig10_ESM.jpg]
