# Supplementary material for: Nucleus segmentation across imaging experiments: the 2018 Data Science Bowl
Source: Nat Methods. 2019 Oct 21;16(12):1247–53. doi: 10.1038/s41592-019-0612-7 (PMC6919559; doi:10.1038/s41592-019-0612-7)
Supplement: Supplementary file 8 — Supplementary Figs. 1–7, Data Science Bowl Strategy Document and Supplementary Material. [file 41592_2019_612_MOESM1_ESM.pdf]

nature methods
SUPPLEMENTARY INFORMATION
https://doi.org/10.1038/s41592-019-0612-7
OPEN

In the format provided by the authors and unedited.

Nucleus segmentation across imaging experiments: the 2018 Data Science Bowl

Juan C. Caicedo[1], Allen Goodman[1], Kyle W. Karhohs[1], Beth A. Cimini[1], Jeanelle Ackerman[1], Marzieh Haghighi[1], CherKeng Heng[2], Tim Becker[1], Minh Doan[1], Claire McQuin[1], Mohammad Rohban[1], Shantanu Singh[1] and Anne E. Carpenter[1*]

[1]Broad Institute of MIT and Harvard, Cambridge, MA, USA. [2]Unaffiliated: CherKeng Heng. *e-mail: anne@broadinstitute.org

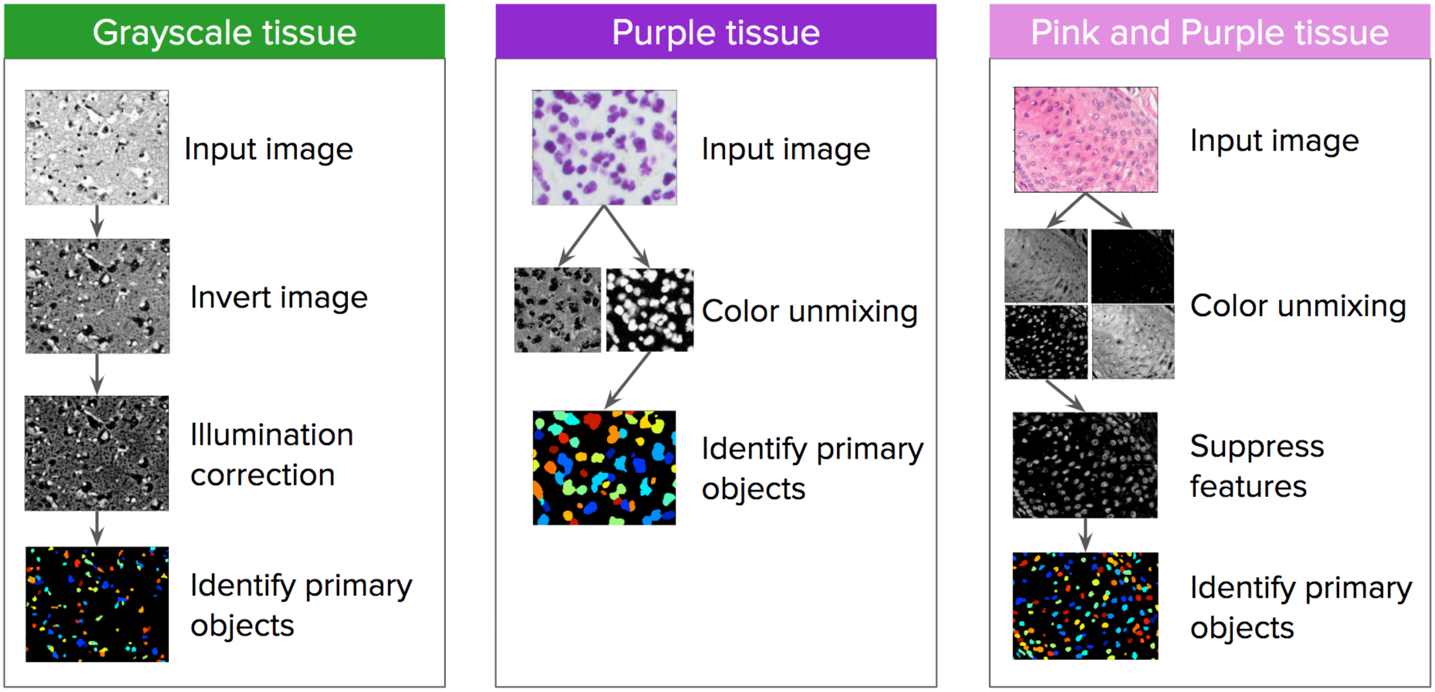

Supplementary Figure 1

Computational graph of the pipelines used for three groups of images: grayscale tissue, purple tissue, and pink and purple tissue

All pipelines finish with the Identify Primary Objects (IPO) module, which runs thresholding, distance transforms and watershed on the grayscale image produced in the immediately previous step. The modules before IPO aim to transform the input image into a grayscale matrix suitable for segmentation.

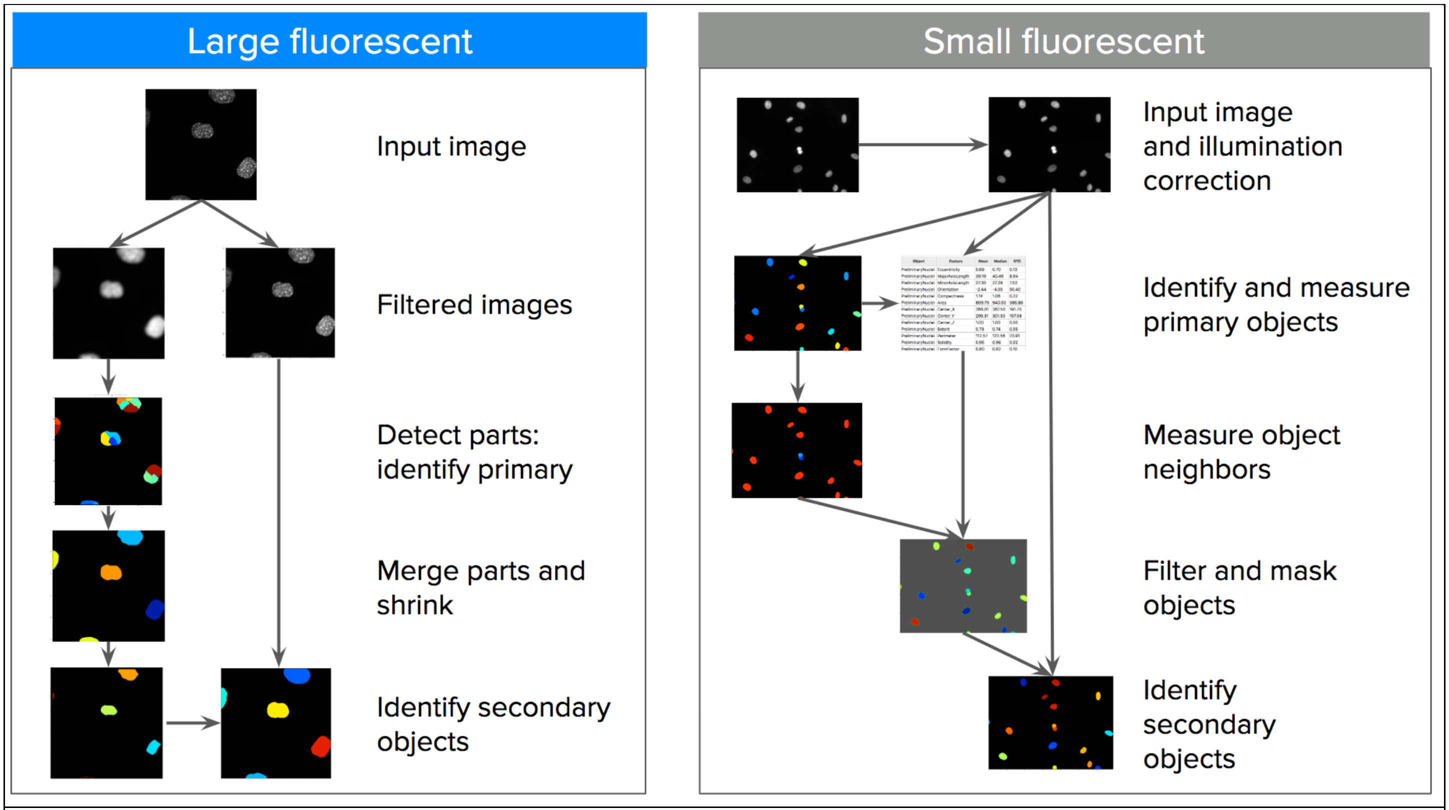

Supplementary Figure 2

Computational graph of the pipelines used for the two fluorescent groups of images.

Both pipelines make use of the Identify Primary Objects (IPO) module as well as the Identify Secondary Objects (ISO) module. Both modules are based on thresholding and watershed using seeds computed from distance transforms and previously identified objects. These were needed due to the large variability of experiments and nucleus phenotypes present in the data. Other modules aim to reduce and filter noise to prepare the image for segmentation.

a

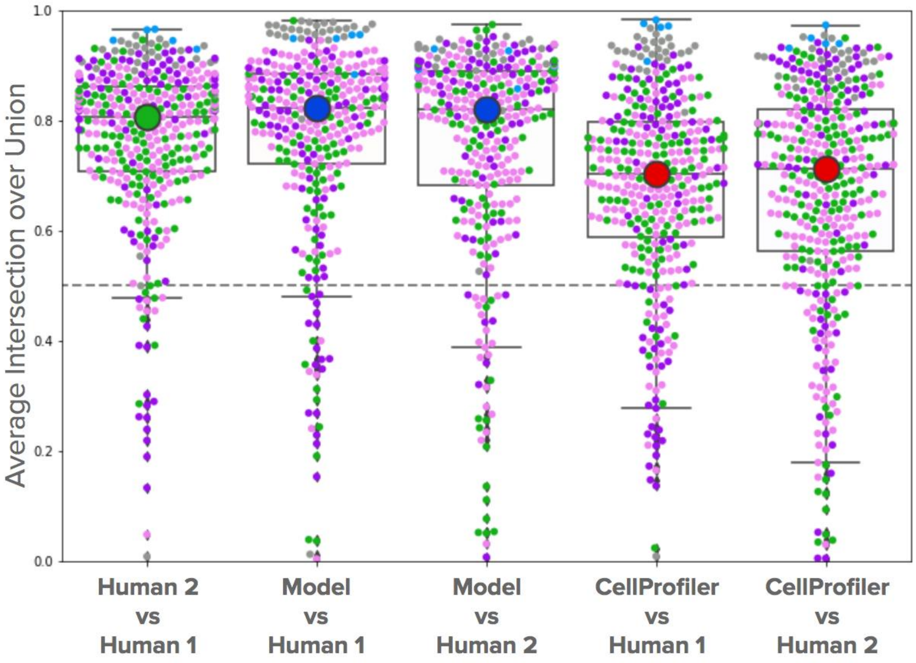

b

Comparing	IoU	F1@0.7
Human 2 vs Human 1	0.806	0.805
Model vs Human 1	0.822	0.801
Model vs Human 2	0.821	0.768
CellProfiler vs Human 1	0.704	0.670
CellProfiler vs Human 2	0.713	0.663

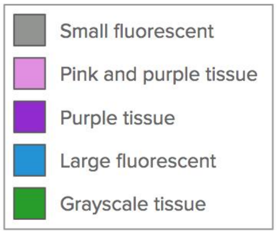

Supplementary Figure 3

Estimation of inter-observer variability.

a) Each small point in the plot corresponds to one object. The y axis reports the intersection-over-union (IoU) score between compared objects and the x axis reports the pairs of subjects or methods being compared. Large points are the median of all object scores. The color of small points corresponds to the type of image the object comes from (legend in the bottom-right). Nuclei from grayscale tissue images is harder to segment for computational methods, while human annotators generally agree on their masks. Purple points display disagreement more often, regardless of the pairs being compared. b) pairs being compared with measurements of agreement in overlap (IoU) and accuracy (F1-score @ 0.7 IoU). Human annotators (green row) reach high object overlap agreement, but the top model (blue rows) agrees more often with both humans than what they agree between themselves. However, the model has slightly more disagreement with humans in terms of accuracy, which means the model misses a few objects more frequently than humans do. The CellProfiler reference displays substantial disagreement with humans in terms of overlap and accuracy.

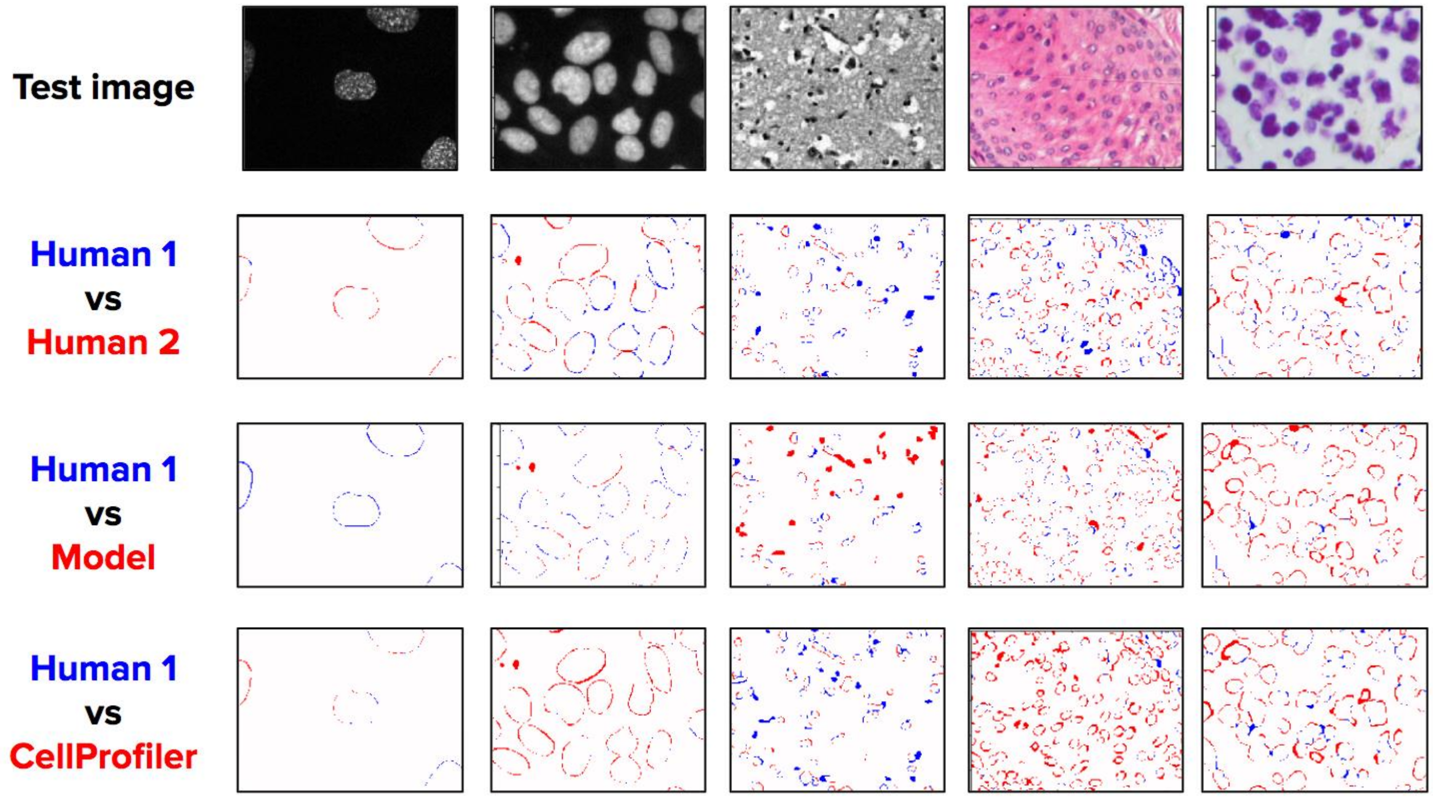

Supplementary Figure 4

Differences in image annotations in 5 selected image examples.

Top row: original images. Next rows: comparison of segmentation between two subjects or methods, one in blue and the other in red. Blue and red objects or outlines indicate annotations introduced by the corresponding observer that do not match the objects or outlines made by the other observer. Ideally, the map should be completely white, which would mean that all annotations are perfectly aligned.

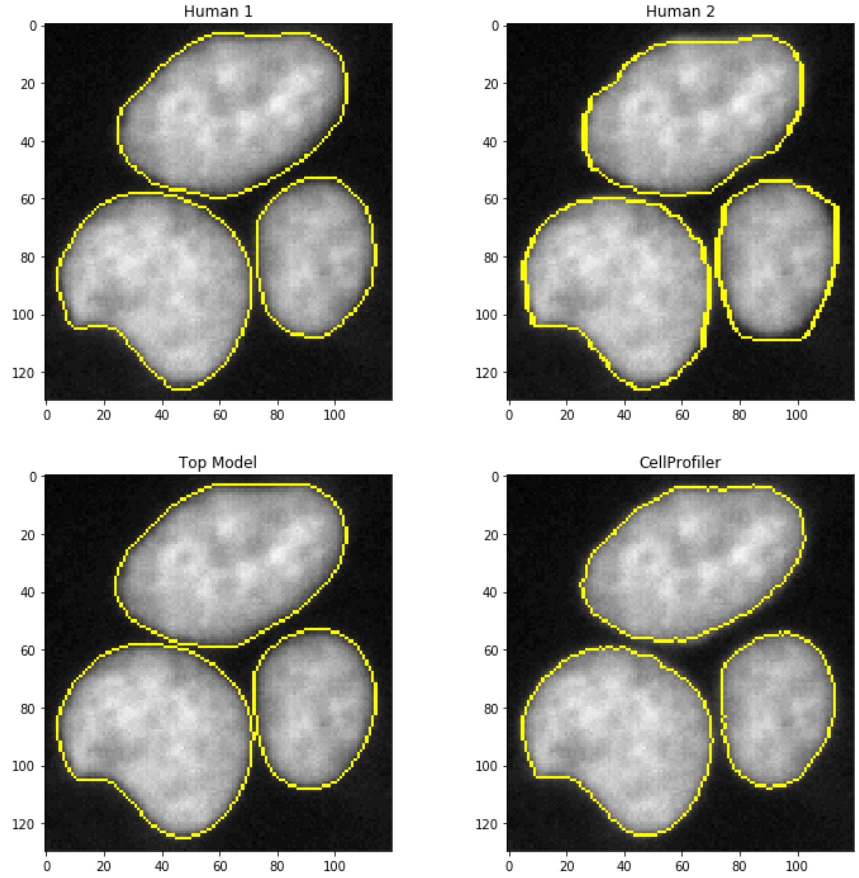

Supplementary Figure 5

Differences in boundaries between annotators and models.

Human annotations introduce subjective noise in the boundaries. The top model learned to produce smooth curves that are close to the edge of the object. This example illustrates how the top model’s boundaries can agree more often with both annotators, while humans may disagree on small boundary details. The CellProfiler segmentations, produced with the Watershed algorithm, fit low-level intensity signal that is not as smooth.

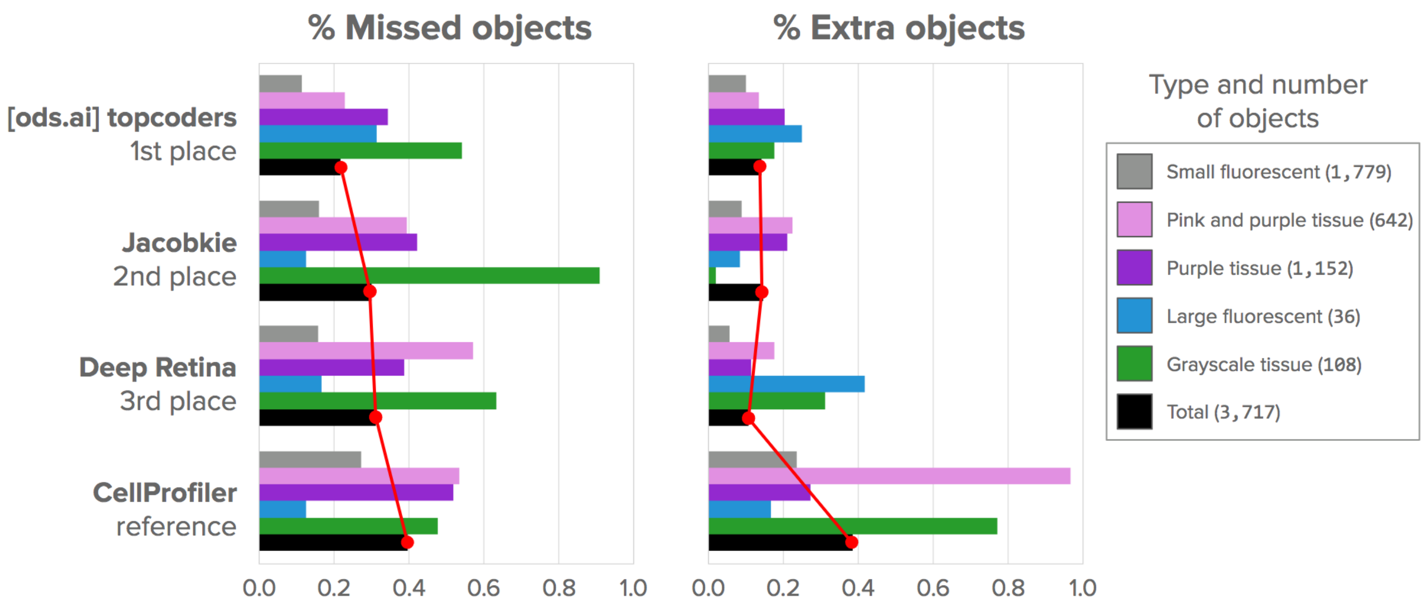

Supplementary Figure 6

Comparison of error rates of the three best performing solutions along with the CellProfiler reference segmentations.

The black bars and red dots show global performance for each participant, while color bars indicate performance for each of the visually distinctive groups in the image collection.

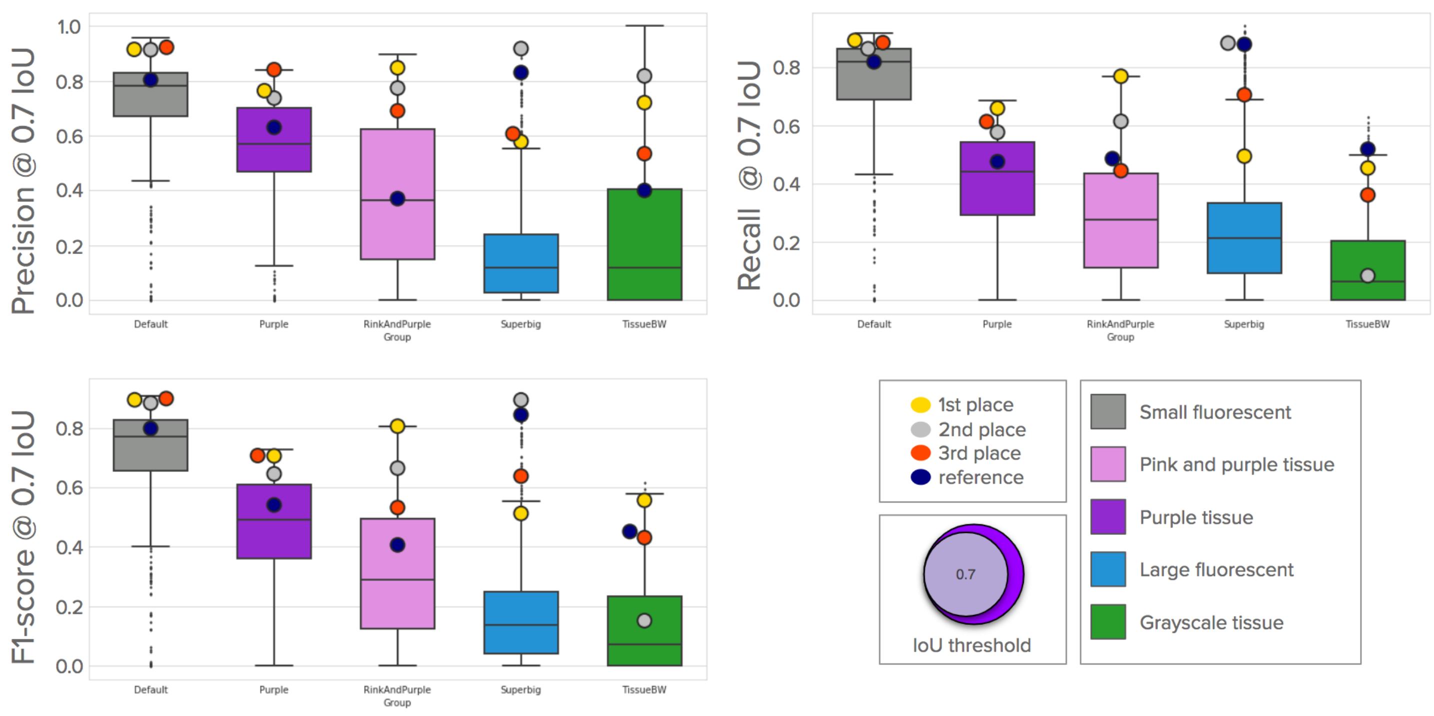

Supplementary Figure 7

Distribution of precision, recall and F1 scores obtained by all the 739 participants in the second stage evaluation, discriminated by image type.

Points in the plot correspond to the top three participants and the CellProfiler segmentation reference. All metrics are measured at the 0.7 intersection over union threshold, which can be interpreted as the requirement that objects have to overlap symmetrically at least with 70% of their area, to be counted as a true positive.

Data Science Bowl Strategy

This file contains informal notes made by the project team during the annotation and implementation for the challenge. It is important to note that this living document was not necessarily kept up to date with final decisions, so it should be interpreted with caution, and is provided in case certain notes may be helpful in understanding the data set.

Table of contents

- Image Preparation Workflow overview: for competition
- Define the biological scope (Broad, done)
  - Any images where nuclei are visible, not just DNA stain only
  - Range of image/nuclei types to be addressed
  - Image sizes
- Design process for annotating (Broad, done)
- Decide how to normalize images for display in annotation tool (Broad, done)
- Determine guidelines for annotating nuclei (Broad, done)
    - Dividing nuclei are 2 nuclei after DNA separation
    - “Holes” within a nuclei will be annotated as part of the nucleus, i.e. they will not be labeled as background.
    - Micronuclei are considered an independent nucleus
    - Nuclei at the edges of images will be annotated and scored
    - Dim, bright, or out of focus nuclei will be included
    - Overlapping nuclei
    - Unannotated DNA stained fragments
- Gather annotation equipment (Broad, done)
- Create annotation tool (Broad, done)
- Refine annotation tool (Broad, done)
- Decide how many annotations are needed (waiting to see how baselines look)
- Devise sampling strategy (Broad, done)
- Gather images (Broad, ongoing)
- Prepare images for annotation (Broad, ongoing)
- Get image sets loaded into annotation tool or distributed to those doing freehand outlines (Broad, ongoing)

Annotate! (Broad, ongoing)
We will not collect multiple annotations of the same images
Expert biologist check over annotations (Broad, not yet begun)
Post-process manually annotated images into the right format (Broad, ongoing)
For already-annotated public sets, convert ground truth into proper format (Kaggle/BAH?)
Choose the competition metric (Allen + Will + Peter, in progress)
Decide what metadata to include (to discuss)
Decide guidelines for selecting images and partitioning into training/validation/test
Stage 1
Stage 2
Take reasonable measures to keep test set away from internet/small circle of people
Do we want to poison the test set? (to discuss)
Prepare images for competition (Kaggle/BAH)
Setup and test baseline methods (Kaggle/BAH, but Broad could help if needed?)
Decided who can compete on the leaderboard vs. cash prizes (done)

Image Preparation Workflow overview: for competition

Note that several sections of this document have details for various steps!

Steps	Automation	Who?
**Obtain images and CC0 release from creators**	**No**	**Broad**
[**OUTPUT**]<br>1. Images of nuclei in native format and unprocessed<br>2. Google spreadsheet of metadata about the image	**---**	
**Separate multi-channel images into separate images**. Multi-channel images from highly multiplexed microscopy methods can exceed the 4 color format of RGBA PNGs. Therefore, each channel, normally represented as a grayscale image, will be saved as its own RGBA PNG, so we	**What is the script called that does these next steps? (can put all steps**	**Broad**

can ensure consistency in the representation of multi-channel image sets regardless of the number of channels. However, there is an exception for natively RGB images typical of color cameras used image tissue sections. These natively color images will remain color and saved as RGBA PNG.	**into one row if it's all the same script)**	
**Global rescaling of image intensities to use full image format bit-depth.**		**Broad**
**Convert to 8 bit-depth**		**Broad**
**Crop to size that has ~100 cells per view** (to avoid sampling too many highly similar cells and to make annotation more manageable.		**Broad**
**Save each image as RGBA PNG with alpha = true**.		**Broad**
[**OUTPUT**]<br>1. 8-bit RBGA PNG, 1 image per channel.<br>2. Supplemental images of other channels, as visual aids for annotation (will not be provided for competition).	**---**	
**Annotate image sets semi-manually** such as with annotation tool (or manually, using images from above)	**Annotation tool or GIMP**	**Broad**
[**OUTPUT**]<br>Annotation label images. (4-color label matrix if annotation tool; outlines if manually annotated)<br>Note: Annotation images will have the same name as the image they reference and will reside in a mirrored folder structure.	**---**	
**Create object masks from annotation label images** (if 4-color annotation image from semi-automated annotation)	**CellProfiler pipeline: Make_objects_from_annotation_tool.cppipe**	**Broad**
**Create object masks from annotation label images** (if manual outline annotated image)	**CellProfiler pipeline: Make_objects_from_outlines.cppipe**	**Broad**

[**OUTPUT**]<br>A new set of mask images is created with a single image for each annotated object.	**---**	
**Convert the resulting TIFF image to an 8-bit PNG** to reduce the file size by approximately 500 fold (for CP 3.0.0 the default mask image is an uncompressed TIFF that takes up an undesirable amount of disk space.	**python script: annotation_conversion_tiff_to_png.ipynb**	**Broad**
[**OUTPUT**]<br>A set of 8-bit PNG images, 1 per object.	**---**	
**Randomly flip and/or rotate images and their annotations** so that competitors can't count on borders always being a certain direction (this is due to the pipeline used to convert manual images)	**Not yet implemented**	**Broad**
[**OUTPUT**]<br>A set of 8-bit PNG images, 1 per object (where the outlines are randomly biased in different directions).	**---**	
**Check that resulting image and annotation/object masks are same dimensions** (because sometimes we crop, it's worth doublechecking)	**Not yet implemented**	**Broad**
[**OUTPUT**]<br>1. A set of 8-bit PNG images, 1 per object.<br>2. 8-bit RBGA PNG, 1 image per channel.<br>These are the images to be provided to Kaggle/BAH to prepare for competition release<br>Note: Object images will reside in a folder with the same name as the image they reference.	**---**	
**Scrub metadata** and assign uuid to object masks and 8-bit PNG images (see "Prepare images for competition" section for details).		**Kaggle/BAH**
[**OUTPUT**]<br>Competition ready images:<br>1. a set of 8-bit RGBA PNG, 1 image per channel<br>2. a set of 8-bit PNG images, 1 per object	**---**	

Define the biological scope (Broad, done)

Any images where nuclei are visible, not just DNA stain only

- We decided a solution should be able to infer the location of nuclei even when a DNA channel is unavailable (vs only images where DNA is directly stained).
  - When the number of fluorescent channels in a typical imaging experiment is limited, freeing a channel by eliminating the need for a DNA stain would be attractive.
- Samples can be generated using fluorescent or absorbance-based stains (we will NOT invert the latter, so nuclei might be light on a dark background or vice versa).
- We could do annotations of nuclei once, and include all Cell Painting stain images (individual channels) in the training sets.
- Always black background? No, would be nice to have flexibility of feeding in whatever color scheme is natural, unless it makes the problem too hard.
- Images will always be RGB (grayscale will just be converted to that)
- Exclude unstained brightfield imaging, though. Existing studies indicate it's a much harder problem - can expand to that in the future.

Range of image/nuclei types to be addressed

We are limiting the challenge to nuclei (from any species) that generally "look like nuclei". Sometimes cells have been challenged in an experiment to the point that their nuclei look so different from typical (lack of structure, extreme clumping/fusion, or unusual sizes and shapes) we ought to exclude them from the challenge (because it makes the problem too hard and because it's not obvious to biologists how to segment them).

We are focusing on cultured cells, tissues, and organisms. We are excluding yeast because they differ substantially from higher organisms in terms of the appearance of their nuclei.

Image sizes

- Contestants will be told the maximum size of the image the final solution must handle (based on our test set).
- Rationale: large images can cause memory problems and compute time issues.

Design process for annotating (Broad, done)

- Allen & Kyle have good strategy for using annotation tool
- Anne has good strategy for using GIMP (with mouse or tablet)

Decide how to normalize images for display in annotation tool (Broad, done)

(See also the section on Prepare images for annotation which contains the detailed workflow).

- The raw images of nuclei typically do not display well on computer monitors.
- Images must be normalized to effectively be displayed.
  - Normalization encompasses any transformation to the intensity information within an image. A common technique is linear rescaling of the intensities to fill the image container bit-depth.
- While easily viewing images is important, normalization can bias the annotations by enhancing or obscuring the boundaries between nuclei.
  - Ideally, the annotation tool would provide the ability to adjust the brightness and contrast of an image, so that the annotator has control over the display of the image.
  - Without these tools, we are normalizing images using our best judgement. While we are aware that the normalization we choose will influence an annotator's decision, we believe display differences will only lead to subtle differences between annotations.
    - We believe the greater source of differences will be an annotator's individual preference or belief in determining the boundaries of nuclei.
      - To account for this source of heterogeneity we will be using several annotators, so that collectively a set of annotations will reflect the diversity in opinion between biologists.

Determine guidelines for annotating nuclei (Broad, done)

- There will be disagreement about annotations, even among expert biologists
  - A major source of disagreement is deciding what is and what is not a boundary between two or more nuclei. When cells are "sick" the morphology of a nucleus can change drastically in terms of shape and size.
    - An example is BBBC002. The ground truth shows there is consistent disagreement between the two annotators when the cell count is small. One annotator reliably sees more nuclei than the others.

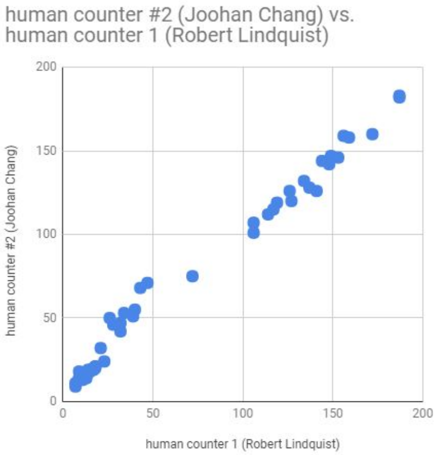

Dividing nuclei are 2 nuclei after DNA separation

- When does 1 dividing nucleus become 2 nuclei (or 1 cell become 2 cells)? It is a continuous process
- We want the solutions to work using only a DNA stain, a common situation. With only DNA stained, it is difficult to discern:
  - Two recently divided daughter cells versus a cell undergoing cytokinesis where the nuclear membrane has already reformed.
  - A multinucleated, e.g. binucleated, cell from a cell undergoing cytokinesis.
  - A telophase cell from a cell undergoing cytokinesis or recently divided.
- Regardless of the number of cells we can discern the number of nuclei.
- Image analysis segmentation favors physically separated objects.
- Therefore, we will consider 2 nuclei to exist once there is clear separation of DNA in a dividing cell, presumably during anaphase.
  - Prior to and during metaphase, when DNA is aligned, an object will be considered 1 nucleus.
- With this approach, the resulting models will identify individual segments of DNA; the biologist can reunite them, if appropriate, using heuristics and classical image processing procedures if needed

“Holes” within a nuclei will be annotated as part of the nucleus, i.e. they will ***not*** be labeled as background.

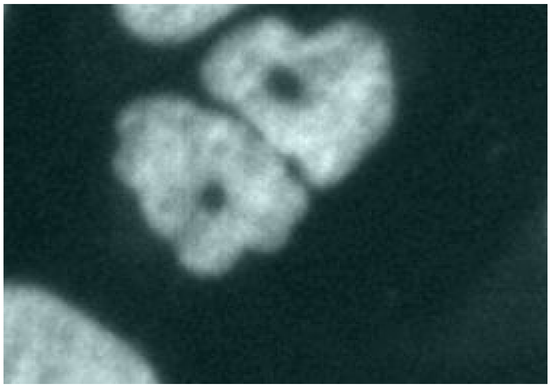

Micronuclei are considered an independent nucleus

- Micronuclei are a common byproduct of DNA damage and disrupted cell division. Since micronuclei are much smaller than a nucleus, and often physically contact the nucleus, are micronuclei still part of the nucleus?
- Micronuclei can vary widely in size and number
- Sometimes a mother nucleus is not apparent
- Micronuclei don’t necessarily contact each other or the mother nucleus.
- Given the above examples of variability in appearance, it has been decided to identify each micronuclei as an independent object.
    - This way, questions related to counting nuclei and relating nuclei to unique cells can be subsequently answered in a separate analysis.
    - Including micronuclei in the same class of objects as nuclei means these objects can span orders of magnitude in size in the same image.
- We will include all DNA fragments that appear to be membrane-bound, but not DNA fragments/debris that do not appear to be membrane-bound.

Nuclei at the edges of images will be annotated and scored

Nuclei at the edges of an image are cut-off/obscured by the edge. Should we exclude edge nuclei from scoring? (and tell competitors we are doing so?) Or even better, should we remove edge nuclei from annotations altogether so we don’t even need to bother telling competitors about this caveat?

- Anne: We may get better models for the main task if we ignore edge nuclei (which is a nice-to-have but not critical).
- Peter: I tend to agree here and i think the main task will likely scale so if we run the algorithm on non-cropped images, all of the nuclei that were edge nuclei in the competition are now non-edge nuclei
- Kyle: Should this also be left to the discretion of the competitors? Competitors could themselves remove the edge nuclei from the training set. In addition, is there a great difference between cropping an image and the field of view a biologist chooses when

acquiring images at a microscope? In many cases edge nuclei are unavoidable and just a part of microscopy. That said, cropping images will lead to a greater number of perimeter pixels and therefore a greater number of edge cells.

- Anne: right, (nearly) all images, whether cropped or not, will show edge nuclei. The microscopy is usually done on an endless sample of cells - there is no way to capture images that do NOT have some on the edges.
- Peter: if we tell competitors they won't be scored than we can certainly leave it up to competitors to remove them. If we do it then it might level the playing field and allow us to focus on the best algorithm vs the team that had more time to do pre-processing of data. I agree completely on the field of view of the microscope, my main thought is that for HCS studies and research, i would expect the researcher to focus on the nuclei in the image not on the fringe because if those nuclei on the fringe are so important I would argue they need to adjust the microscope
- Anne: yeah, although it would be nicest to have solutions that identify the edge nuclei, biologists usually don't measure the cells on the edges because they are partial. I agree we want to simplify things for competitors to level the playing field.
- Kyle: I'll create the process to remove the edge cells. We can skip this step if we change our minds. We'll need to add this to the image preparation workflow table
- Anne: Let’s proceed. However, let’s still have annotators mark those nuclei so that in the future we can train models to recognize them as well.
- Juan: I think leaving them in or out probably makes no difference with respect to the models. This is something that should be decided by the user of the model, instead of purposefully being ignored by the model. Suppose we decide to leave edge nuclei out of the test set, and therefore these are not scored in the competition. Does it mean competitors can keep or remove edge nuclei in their test set solutions, and we guarantee either way their score will be the same? Or does it mean they have to remove edge nuclei themselves because if they leave them, their score will be penalized? Judging by how performance metrics usually work, I think it is the latter case. It's cleaner to ask for all visible nuclei, independently if they are truncated, overlapping, in the edge, and so on. Edge nuclei is just a special case of visible nuclei.
- 11/13 Phone check-in: the DSB group is okay with leaving the border nuclei annotated. Cropping images is okay even if this introduces additional border nuclei. Juan’s reasoning regarding clarity resonated with the group.
- Anne is excited this seems possible, it will certainly generate the best, most usable models!

Dim, bright, or out of focus nuclei will be included

The following types of nuclei will be included. In general, ambiguous nuclei-like objects should be included in the annotation.

- Sometimes the DNA staining is not strong and while it is above background, the intensity is not as clear as the majority of cells.
- Out of focus cells will appear smooth and blurry. The intensity can be lower than an in-focus cells.
- Sometimes DNA condenses and a nucleus will appear much brighter than an interphase

cell. The cell could be entering prophase or apoptosis.

Overlapping nuclei

- In tissues (less frequently in monolayers) two or more nuclei will overlap.
- By eye overlapping nuclei can be distinguished, but it is impossible to encode the boundary of each individual nuclei in a single annotation image.
- Peter: is it important to distinguish between dividing cells and overlapping cells?
- Anne: dividing nuclei usually do not overlap; their appearance is pretty different from overlapping nuclei. so it doesn't present a unique problem to the annotation scheme.
- A line could be drawn between each nucleus
    - However, this line is a compromise that doesn’t reflect the true boundary of either nucleus.
    - If the overlap is significant, the lesser nucleus would appear greatly deformed.
- Two annotation images could be presented, each image would have the correct annotation for one (or more) overlapping nuclei.
    - Peter: in the test set of images Kyle sent there was a single image mask per nuclei, i'm assuming that these masks are build from a single annotated image and to correctly represent overlapping nuclei you'd need a separate annotated image for cells that overlap so that one nuclei is correctly depicted in image one and the other overlapping nuclei is depicted in the second image.
- Our final decision: The uppermost cell wins. Whatever cell is on top should be labeled properly. This way at least one of the nuclei is true even if it is at the expense of the other. The other nucleus (or its fragments) should then be labeled even if misshapen.

Unannotated DNA stained fragments

Sometimes objects will be stained for DNA, but are not necessarily nuclei or even DNA.

Contextual clues indicate this. Is this the remains of a dead cell, an aggregate of the stain, non-specific staining of debris? In any case, if the object under question deviates too far from what would be considered a nucleus, membrane-bound fragment, or a recognized state of a distressed nucleus (i.e. micronuclei) then these objects will be labeled as background.

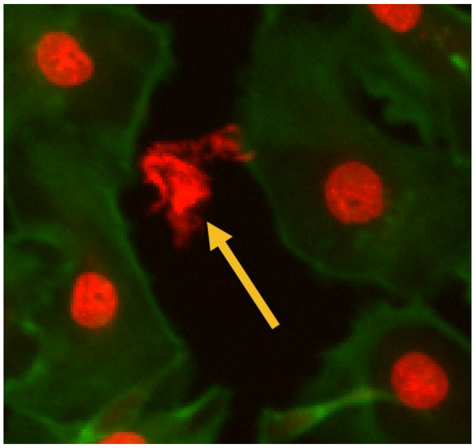

Gather annotation equipment (Broad, done)

Anne & Kyle procured a Surface Pro 3 + pen + charger and it's working well
Jeanelle will order an Apple iPad Pro + pen for arrival ~10/16

Create annotation tool (Broad, done)

- https://github.com/broadinstitute/annotate
- Requires python installation
- Runs a web server
  - From the cli `python -m SimpleHTTPServer`
  - From a browser `localhost:8000`
- Alternatively, an image set can be prepared for annotation over the web
- Currently resides in a private Github repo (the annotation server is being hosted on GitHub, which is tied to the annotation tool repository)

Refine annotation tool (Broad, done)

Allen added features to meet peoples' needs as we've begun annotating

Decide how many annotations are needed (waiting to see how baselines look)

- It is difficult to know how many annotations will be necessary without building a solution, because there is not a general rule that relates the number of annotations and the success of a model.
- The Imaging Platform has had success with few annotations, but also situations requiring many annotations. And we have never tried robustness across very different experimental setups

    - 5 images, nuclei segmentation, Victor, BBBC023
    - 200 images, nuclei segmentation, Jon and Allen, label images, plateaus after 50
    - 1000 images, blood cell classification, Jane, bounding boxes
- Our image sets vary widely in terms of image number. To start we will annotate 5-20 images from each dataset. (2000+ cells per sample)
    - Tissue images will be an exception if the input is a stitched image. Though we can provide unstitched images, too. Better to use many small pieces of different images than annotating one huge image entirely.
- We can run baseline accuracy with Jon and Jane's architecture (see later step where we run baselines)

Devise sampling strategy (Broad, done)

By default sampling will be from a uniform distribution. However, it is common to have image sets where a uniform random sampling approach would require annotating too many "easy" nuclei. This would lead to a training set that is diluted of the nuclei that are most challenging to segment and that motivate this competition; we want to choose a variety of images, not a lot that all look the same Therefore, we have implemented an additional sampling strategy that uniformly samples from a particular attribute or metric whose distribution represents the different classes of nuclei within the image set. For example, we sampled one image set using CellProfiler nuclei counts as follows:

1. Cell count for each image is produced by CellProfiler segmentation. Difficult to segment objects should lead to over- or under-segmentation.
2. Stratify the distribution of cell counts (assuming variation is derived from segmentation errors as opposed to seeding). In other words, divide the distribution of cell counts into bins.
3. Take images from each bin defined by the stratification in a uniform manner.

The sampling method described above can be generalized to other aspects of the nuclei we can measure. The method will be weighted towards difficult to segment images. The specifics of the sampling method used for any image set can all be traced back to the scripts that process each image set.

For 3D images, the annotations will refer to the image in the stack that is most infocus. The focus is determined using Additional images in the stack will be provided the same as if they were different fluorescence channels of an image sets.

Gather images (Broad, ongoing)

Sources:

- Images from our collaborators on Broad's servers and AWS (Kyle emailed many of them and asked permissions)
    - Go through project folders in the imaging platform and the cold bucket, and add to spreadsheet if there are images
    - Assay devs should go through and Yes/No to project to be sure it's ok/polite to

        ask for images
    - Add point person/ contact to the spreadsheet
- BBBC image sets some of which are already annotated (public, will only be used for the training set, not test)
- Emailed grad school buddies for 1-2 images
- Posted to CellProfiler forum requesting 1-2 images to get diversity, submitted via a Google form
- Wikimedia Commons (for training set only, since these are already public): Anne took a quick skim, it does not seem to have a ton of appropriate images unless she just isn't checking the right categories. I looked for fluorescence images, I'm sure we could get a bunch more tissue images - didn't search for those yet. Here are the ones she found that look good:
    - CC Attribution-Share Alike 3.0 Unported
        - https://commons.wikimedia.org/wiki/File:Rat_hippocampus_stained_with_antibody_to_NeuN_(green),_myelin_basic_protein_(red)_and_DNA_(blue).jpg
        - https://commons.wikimedia.org/wiki/File:Culture_of_rat_brain_cells_stained_with_antibody_to_MAP2_(green),_Neurofilament_(red)_and_DNA_(blue).jpg
        - https://commons.wikimedia.org/wiki/File:DAPI_%CE%B2_cells_of_pancreas_1.jpg
        - https://commons.wikimedia.org/wiki/File:DAPI_%CE%B2_cells_of_pancreas_2.jpg
        - https://commons.wikimedia.org/wiki/File:Epithelial-cells.jpg
        - https://commons.wikimedia.org/wiki/File:Microscopic_image_of_stem_cells,_Hues_9_stained_with_DAPI.png
        - https://commons.wikimedia.org/wiki/File:Caenorhabditis_elegans_DAPI.jpg
    - Creative Commons Attribution-Share Alike 4.0 International
        - https://commons.wikimedia.org/wiki/File:Ki67-Tubulin.jpg
        - https://commons.wikimedia.org/wiki/File:Sequential_sections_of_a_colon_crypt_showing_normal_high_expression_of_PMS2_(A),_ERCC1_(B)_and_ERCC4_(C).jpg.tif
        - https://commons.wikimedia.org/wiki/File:Pig_oocyte_dapi_4.jpg
        - https://commons.wikimedia.org/wiki/File:Pig_oocyte_dapi_3.jpg
    - someone could gather these images and prep for annotation
        - +minhdoan@broadinstitute.org Can you please place these images into two Google Drive folders (Kyle can tell you exactly where) according to their license types? And perhaps find a bunch of good tissue images too? These will need to be 100% manually annotated so we don't want them to go through the normal pipeline.
        - sure, just let me know where
        - Cool! See two folders "Wikimedia" in here: https://drive.google.com/open?id=0B2nF3dOKaYvHeDNMcDRzYU1PaW8
        - In the end: Decided to only use public domain images

- Beth is digesting annotated images that are public, so we can reduce the number we need to do ourselves! Check in w Anne/Kyle if any Qs about what is appropriate to include.
    - There is a section of the Image sets for DSB google sheet devoted to these, noted as “already-annotated public set (we think)”

Prepare images for annotation (Broad, ongoing)

Images to be used in the competition and supplemental images used for annotating are processed and created at this stage. For details on how the images to be released in the competition are prepared see the table “Image Preparation Workflow: for competition”.

An additional set of images is created that are not be part of the competition, but are necessary for using the annotation tool. Below is a description of the types of images in this set:

- **blank annotation** or **seeding images**: The annotation tool requires an annotation image to be present before manually annotating. By default a blank image is created for this purpose. Seeding strategies will be pursued on a case by case basis per image set. These will be 8-bit RGBa PNG.
- **normalization images**: The current iteration of the annotation tool does not provide the ability to adjust the display of image to change intensity or contrast. Therefore, a normalized copy of the image is created whose intensities have been stretched to give nuclei a brighter and clearer appearance on a tablet or monitor. These will also be 8-bit RGBa PNG.
- **rgb images**: Another set of images will be created when there are multiple channels. These images will contain 2 or 3 of the channels in a composite color image. These images will 8-bit RGBa PNG. Unlike the normalization images there will not be any rescaling of individual channels. It is recommended to use a tool like FIJI in order to adjust the brightness and contrast of each channel to the individual preferences of an annotator. Note that FIJI does not read the alpha channel of an RGBa PNG, which means the fourth channel stored in the rgb image will be dropped when imported into FIJI. Therefore, the rgb image, even though saved as an RGBa PNG, is effectively just RGB.

Note, these images will have the same name as the image they reference and will reside in a mirrored folder structure.

Get image sets loaded into annotation tool or distributed to those doing freehand outlines (Broad, ongoing)

Kyle & Allen, ongoing

Annotate! (Broad, ongoing)

Whole project team

We will not collect multiple annotations of the same images

This might be worth revisiting after the competition as part of a paper to measure observer variability.

Currently we are producing a single annotated image, by a single person, for each image. None of our data is annotated by multiple people.

- Would we prefer to have more data, or less data but annotated by two different people?
- Peter: When you say annotated by “multiple people” I’m assuming that you’re talking about two people working side-by-side to produce the annotation vs two people separately producing their own annotations that would require them to be merged. (Anne: we are doing neither currently, we were wondering about whether to do the latter)
- If we did have multiple annotations, we could
  - Combine them to create a “consensus” annotation image through some combination of the labels to calculate the probability that a given pixel belongs to a label (but why do this, if we bother to collect two annotations, let’s just provide them both)
  - Or, Provide both and let competitors decide how to handle
- Anne votes that we do NOT collect multiple annotations of the same image. It’s too tedious. Maybe after the competition is launched we can have a second biologist manually annotate the test set just for fun/future use.
- Peter agrees with Anne - I think for this competition it’s better to use single person annotations and favor more data, there could be a follow-on competition to this where the top algorithms are looked at and the training/test sets utilized images where multiple people produce annotations to reduce overall bayes error.
- Juan: It all comes down to how practical it is. Multiple annotations is probably not needed, as nuclei do not have complex shapes that have to be disambiguated by multiple people, as in the case of segmenting trees in natural images. Just to confirm this, an independent annotator can label a random set of images only to measure how much agreement there is with previous annotations. If we are above, say 99% of agreement, then we are done. But this is probably not critical.
- Seems like we all agree this “would be nice” but is not necessary. This might be something to do after the competition.
- 11/13 Phone Check-in: Given the current progress of annotating and curating images we will not provide additional coverage of annotations for the test images. To echo the previous point, it would be nice to have, but not necessary.

Furthermore, having a handful of extra annotations is not meaningful, a sizeable fraction of the image set would be required, which represents a significant time investment. This could be done after the competition to test observer variability.

Expert biologist check over annotations (Broad, not yet begun)

Mark each image when done in the google spreadsheet

Post-process manually annotated images into the right format (Broad, ongoing)

- Claire & Beth & Anne figured out a CellProfiler pipeline to do this
- For 100% manually annotated images, the edges need to be thinned sometimes (and thinned properly: meaning pushing them outwards rather than inwards), and also around the edge of the image we do not have enclosed outlines for those cells.
- Enclosed spaces in between cells are marked in blue
- TODO: Also need to adapt the script to preserve small chunks of DNA in our pipeline for low-res images: Sometimes my outline is around a single pixel, which is thrown out during the pipeline. Such blobs of DNA can be up to 9 pixels total but with only one internal pixel it is deleted by the pipeline. Need to figure out how to keep them in (or restore them).
- Once finalized, we need to apply the pipeline to the annotated images as they are created

For already-annotated public sets, convert ground truth into proper format (Kaggle/BAH?)

Beth is working on vetting these sets which may have annotations in unusual formats that need to be converted into our settled upon standard.

Choose the competition metric (Allen + Will + Peter, in progress)

Splitting and merging of nuclei is the challenging part and should dominate the scoring. We don't want to emphasize precise edges of each nucleus in the score (Otsu thresholding does pretty well at this).

- Paper with options: http://arxiv.org/abs/1502.05082
- Coco metric - basically Average Precision at a threshold of intersection-over-union:

http://cocodataset.org/#detections-eval

- We used it for Jon's project
- It's been used in the past with high impact competitions

- Quality of segmentation + Object identification
  - 90% of nuclei are "easy", so this part of the problem shouldn't weight score heavily.
- Object first, then pixel accuracy per object
- In reality we care about a combination of speed + accuracy. Although speed can often be improved later, is it possible to put at least some limits on compute time by including computational complexity in the scoring metric? Allen suggested maybe the metric penalize for number of parameters in the model
- Allen & Anne chatted ~10-31 and are on the same page so he can decide what metric to use in collaboration with Peter & Will.
- Juan: I suggest to focus only on accuracy aspects for the competition. Number of correctly detected nuclei, and pixel accuracy should be prioritized. The metrics for object detection used in the COCO challenge can be used for these purposes. See this link for more details: http://cocodataset.org/#detections-eval

Decide what metadata to include (to discuss)

We plan to strip ALL information other than pixel intensities from the images themselves, including filenames (see section on breadcrumbs later). Here we are talking about instead the following type of metadata:

- cell type (species, etc.) & stain type
  - There will be a lot of variety here, and images in test set will be in categories not present in the training set. We want the final solution to get this information (if helpful) from the pixels themselves.
  - Anne votes that we do *NOT* include this metadata in the competition (we can provide afterwards in case anyone cares, though).
  - Peter agrees with Anne, no need to disclose otherwise if we do it seems like we're defeating the purpose of building a generalized nuclei finder.
  - Juan's opinion: I generally agree that the metadata can all be left out. The solution should be entirely based on visual clues.
  - There will be different classes of cells, and one strategy would be to train a model for each cell class and then a separate discriminator model to read in the image and determine which cell model to feed the image to. In this case, if different cells were photographed with different systems, there may be a way to discriminate between cells based on images size/aspect ratio/resolution/inferred scale. If that is true, people may end up sorting photographs by those factors in order to determine which cell model to use. To your point, if the final validation set uses different microscopy setups, then the people that use that strategy won't do well at all in the final scoring, but the early score board and models will be swamped with people building a cell sorter that relies on useless information. Counterpoint: some of that metadata IS useful (IS fair game). We'd be happy with a solution

that figured out the resolution of the microscope used and adjusted accordingly, for example.

  - Something like this happened with the https://www.kaggle.com/c/the-nature-conservancy-fisheries-monitoring competition. People spent a non-trivial time identifying boats and creating algorithms that handle the various camera angles specific to each boat. In the end—those people ended up not doing well on the final scoreboard, but all of the time that was spent on doing well on the public leaderboard was wasted.
  - Our goal here is robustness across totally different data sets, because there are really infinite possible cell types/image types. And, the validation sets that drive the leaderboard will be different from the training sets so that will lend a clue that the strategy won't work - though people may end up optimizing to whatever particular examples we have in the validation set and those won't work well in the final testing.
  - We could double check that if one creates a classifier for the cell types on the training set based on the features I mentioned that it either 1.) does not work or 2.) works well in the training set but not the test set. (But this is a lot of work) If that's the case, then we should be fine to simply be explicit about the fact that in order to do well in the scoring phase, the teams cannot rely on artifacts related to the image acquisition details.

- Manually annotated vs. assisted with automation?
  - ~90% of training images will be assisted with automation while 10% will be completely manually annotated.
  - Anne thinks it is important to provide this information to the competitors because the manual annotations will be higher quality than the automation-assisted and because the test set is 100% manual. Telling them this information allows them to use the large assisted set for training but fine tune using the manual set. Thus the resulting model will be more accurate (per the test set, but also for the real applications).
  - Peter: I agree with Anne's arguments but feel like it largely depends on bayes error for automation-assisted vs hand labeled images. I'm not sure I understand what kind of accuracy we're talking about with regards to manual vs automation-assisted. Is the accuracy relative to the nuclei outline or whether or how accurate the annotations will be in even identifying nuclei and the automation assisted one might miss some nuclei all together. If we provide them this information i would assume competitors will split the 10% of hand labeled images and use a small portion for training and a much larger portion for their dev set. Overall I think my answer depends on the accuracy difference. If it's large then yes, i think we can give them the two labeled sets and indicate that the dev and test sets for the leaderboard will be 100% hand labeled. Otherwise I'm really leaning towards not providing this metadata.
  - Anne: Hm, so far we have only a single image annotated in both ways. Do you think it's important to create a larger set annotated in both ways to assess this? I would assume that the leading teams' accuracies will be within fractions of a percent of each other, such that any difference from automated to manual will seem large (see other section on whether to have multiple annotators where we cover this topic).

- Who is the annotator?
    - The training images will be annotated by an assortment of people, around 6-8
    - All of the test images will be annotated by one of 2 biologists
    - Anne doesn't have a preference about this; it could be useful in case one of the automation-assisted annotators is not matching a person's model well, they might choose to ignore that part of the training set. In her opinion knowing the person does not add a ton of info as compared to the prior question (manually annotated vs assisted)
    - Peter: I agree it doesn't add a lot of info providing annotator, i'm in favor of not providing this. The less info we provide i think will hopefully lead to more generalized and simple models.
- 11/13 Phone check-in: To promote the generality of the solutions, all metadata will be removed from the training set. This includes scrambling the names of the images with a salted-hash and removing the directory structure. Also, any metadata regarding the creation of the annotation images will be removed. The idea is for the challenge to promote the pursuit of the ideal solution: given any biomedical image with nuclei, produce an accurate segmentation. For example, imagine dragging an image into a window and a segmented image is returned. The more metadata we provide for the competition the more likely a solution will rely upon a specific piece of metadata that might limit the implementation of this solution later on. The metadata will be preserved for studying and assessing the performance of the solutions and the competition itself after the competition ends.
- Anne: I'd like to chat at our next checkin. Our decision depends on how much difference we expect between 100% manual and auto-assisted annotations. If the former is much more accurate than the latter then I think it makes sense to include this metadata. It's in essence providing a large set of messy ground truth and a small set of very accurate ground truth (and the test set would all be the very accurate 100% manual style). The goal would be to allow more accurate solutions to be made, not to help people build models that rely on metadata per se.

Decide guidelines for selecting images and partitioning into training/validation/test

Kaggle/BAH recommend a two-stage setup:

Stage 1

Give competitors:

- **training set** (20 experiments x 10 images each; TOTAL: ~200 images, ~20,000 cells), ~90% generated via automation assistance, 10% hand annotated
- **validation set** (15 images, each from a different experiment; TOTAL: 15 images, ~1500 cells), 100% hand annotated
- all images will be provided with accompanying **annotations** (ground truth).

Notes:

- Purpose of validation set is to allow competitors to submit results on it to the leaderboard
- Of course, it's easy for them to cheat on the validation set and submit perfect results (either annotating by hand or just submitting the provided annotations as their results), so the leaderboard is something of an honor system and likely won't predict well who will win
- At end of stage 1, competitors must submit their model (including weights) somewhere secure
- Aiming for all these images to be licensed CC0 so anyone can use for anything going forward
- It's ok for this data to have been public already. Worst downside of that is that it means they can find where it came from then hand-annotate a bunch more data from that source, but since none of validation/test images come from those sources it doesn't help them to do so (vs. grabbing any random images they can find).
- If necessary, these images could come from public sources such as IDR or The Cell Image Library (esp for validation set where we need a variety).
  - Downside is they are not CC0 - so we need to keep track of attributions

Stage 2

Give competitors:

- The **test set with no annotations** (35 images, ~3500 cells), entirely hand annotated. Give them five days to submit results from it (each image is from a different experiment, plus possibly additional "poison" images)

Notes:

- They could, of course, just hand-annotate and submit those results but hopefully we would figure this out when we look at their code and realize it would not produce those results.
- The top finishers (to be cash prize eligible) must submit a narrative description/technical paper on what strategy they took for us to assess
- IF we can get their system/code running (possibly quite impractical) then we can test it to be sure it produces the same results as submitted. Could also test against a separate holdout set to be sure results aren't crazy bad.
- Stage 2 proposed ~5 days: we could shorten (to ensure reasonable runtime in the solutions) but you run into trouble with teams having personal/medical issues or complaints about how long it takes to download stage 2 data
- We are not worried about cross-contamination between the test and training/validation sets, because each image in test/validation is from a different experiment.
- These should be never-have-been-public. Even if we use non-public images from an image set where the rest is public, some competitors might have grabbed those to use for additional training.

- Among the test images, we need to demonstrate a variety from easy (mammalian cultured cells) to challenging (tissue).
- The test set images will be 100% manually annotated (no superpixel tool, no automated thresholding)
    - One tiny exception: converting outlines to objects involves automated step that can introduce some bias: the pixels between two objects are assigned to the top-most object (I think, could be left-most). We should explain this to competitors but also say that images (all images, including training/validation/test) will be randomly flipped and rotated such that they can't count on borders always being a certain direction.

Take reasonable measures to keep test set away from internet/small circle of people

- We could keep test images completely away from the internet to avoid cheating/hacking, but this is pretty impractical?

- Are reasonable security measures ok?
    - Yes, this really should be fine to use Google Drive/Dropbox (non-public obviously)
    - Smaller group of people is better but we don't need to be really paranoid

Do we want to poison the test set? (to discuss)

We will poison the images. The feeling is that the dataset is small enough that some might consider annotating the test set by hand. The questions that remain is how many poison images to include in the test set. The suggestion is 100 or 1000 poison images.
We might want to poison the test set (with unannotated, unscored images) to make it more tedious to cheat by hand annotation and to encourage reasonable runtimes. But this might provide advantage to big competitors who have unlimited computational resources.

Peter: agrees with Will that we probably don't have to worry too much about cheating and I'd favor not "poisoning" the test set. I honestly defer to Anne and Broad on this since you'll have a good understanding of how easy/quickly 35 images could be annotated if someone was going to attempt to cheat

For reference: 35 images is ~35MB, takes ~35 hours to annotate.

Should we make our "rules" for annotating publicly available? (to assist people making consistent annotations) This has the downside of making it easier to cheat by hand-annotating. Let's not release these.

Prepare images for competition (Kaggle/BAH)

Kyle sent over an image set in the final format to Kaggle/BAH team so they can get started working their magic, to accomplish the following:

Removing breadcrumbs

- **Image metadata**, if not obscured or removed, can be used by competitors to circumvent the image challenge.
  - For example, a winning model might take advantage of the date an image was acquired to tell how related two images might be. The assumption is images acquired closely in time would contain similar objects.
  - Not a huge concern here, though, given that the test images will be from entirely different image sets than the training images.
  - Still, we should randomly flip and rotate images (see notes later about certain steps of annotation being directional)

- **Filenames -** Will - “We like to use a (salted) hash for the competition filenames. If you label images 1:n and find out you need to remove/add an image, you end up having to give everything a new id so there aren't gaps. If you use a hash, you don't have to care about having a clean sequence of ids.” I think these following notes are outdated?
  - Images will be assigned a uuid (or hash? Or is that the same thing?)
    - Python uuid.uuid
  - Annotations will be assigned a uuid
  - A lookup table will map each uuid to the internal organization of the data
- **Create/Modify dates** – images should be all set to have same timestamp
- **Image size/aspect ratio** – Images come in all shapes and sizes. Further, we crop some before annotating. The validation and test sets will not come from the same experimental setup as any training set images. So we are not concerned about competitors learning something about images from their shape and size. Still, we are introducing a rotation/flipping step into the pipeline to randomize this a bit. Likely we should tell competitors what we’ve done so they don’t waste energy doing something that won’t help on the test set?

Annotations: Other Kaggle examples

- Data Science Bowl 2017, Lung cancer CT scans
  - https://www.kaggle.com/c/data-science-bowl-2017
  - Annotations are a diagnosis from a pathologist
  - Binary segmentation
- Fish monitoring
  - https://www.kaggle.com/c/the-nature-conservancy-fisheries-monitoring
  - Annotations are folders that contains images with a certain fish

Setup and test baseline methods (Kaggle/BAH, but Broad could help if needed?)

- To ensure problem is in a good range of solvability. We want accuracy to not be 99% but also not terribly low
- Baseline accuracy with Broad team's (Jon and Jane's) architecture

Decided who can compete on the leaderboard vs. cash prizes (done)

Decision: Broadies (and BAH/NVIDIA, etc.) can compete, but they wouldn't be eligible for prizes. Harvard and MIT would be allowed to fully participate, because their affiliation with the Broad Institute is reasonably separate. The immediate project team (Carpenter lab, Will/Peter, etc.) should not post to the leaderboard.

Supplementary Material

A. Dataset statistics

In the context of this dataset, an experiment is defined as the biological study of a specimen, which includes the physical preparation of a sample for microscopy evaluation, the development of the imaging assay, equipment configuration, and image acquisition. Normally, a biological experiment is conducted in a facility, by a group of researchers, using specific equipment in a bounded time schedule.

The description of all image sets is reported in Supplementary Table 1, which contains quantitative information about images, including resolution, total number of nuclei, SNR and contrast (computed according to Ulman et al. (2017) [1]. It also contains qualitative information such as cell type and visual group assigned based on the optical appearance. In summary, the full dataset comprises 37,333 manually annotated nuclei, 841 images, 31 experiments, 15 cell types, 12 image resolutions, and 5 visually similar groups. The average SNR in all experiments is 2.488 with a standard deviation of 1.697. The 31 experiments were assigned to two partitions, 16 experiments for training and first stage evaluation, the remaining 15 experiments for the second stage evaluation.

We collected only those metadata parameters that were available to all experiments. Not all experiments have detailed metadata information about their development because images were donated from labs all around the world: some of them had metadata available, some others did not, some of them had confidential information that cannot be shared, some others were already publicly available on the web and did not report these properties. Our team of expert biologists curated and annotated the image collection to the best of their ability, and included images that can add value even without complete descriptions.

The most challenging aspect of the dataset is the large experimental variation. For our purposes, we treated the dataset variables as unknown confounders, except for the source laboratory, which is a proxy variable to all other details. We selected a few images from large experiments to avoid over-representation of samples with the same technical variations. In some cases a random sample was used, in other cases, manual selection was performed. We did not balance the dataset based on the five visually distinctive groups of images, as our goal was to introduce technical and experimental diversity to simulate the real world variations that a widely applicable nucleus segmentation method would find in the wild.

B. Description of evaluation metrics

The Intersection-over-Union (IoU) metric is used to estimate object coverage and detect correctly segmented nuclei. There are several types of errors that can be measured at one IoU threshold, including merged, split, missed and extra objects [2] following this procedure: given N

segmented objects and M target objects, we compute the $N \times M$ matrix of IoU scores. Ideally, this is a square and diagonal matrix. However, with extra objects the matrix will have more rows, and with missed objects it will have less rows. Over-segmented objects appear as extra rows with at least two non-zero entries in the corresponding target mask column. We keep the estimated segmentation with maximum IoU for each target mask (only if max-IoU is above a threshold) and the remaining pieces are counted as false positive objects.

The official competition metric looks at true positives, false positives and false negatives at multiple IoU thresholds and computes a precision score as originally suggested by Everingham et al. [3] for semantic segmentation. These scores are averaged across all images and all thresholds. In the study presented in this paper we also computed the F1-score, which is defined as the harmonic average of precision and recall, and is used to compute the accuracy of a test. We use F1-scores to interpret the accuracy results in the context of nucleus segmentation given that there is only one class of objects to be segmented, and the score indicates whether it's correctly segmented or not [2].

All scores are normalized by image, which means that if an image contains significantly more objects than others, this will not affect the results and will make scores comparable from image to image. The scores are not normalized by dataset, image type or experiment. These variables are ignored when computing our aggregated scores for simplicity. During the competition we did not assign more value to certain types of images, all were treated equally. However, in our analysis of the results, we decomposed scores by image type because we observed that this variable drives an important part of the error. This can be observed in Figures 1c, 2a, and 3.

In several analyses run after the competition ended, we used a single IoU threshold to compare performance, estimate error rates, and interpret the results in a more intuitive way. We chose IoU=0.7 as our reference cut-off for all analyses that require a single threshold in this paper. We made this choice for two reasons: 1) it is a central point in the range of thresholds used for evaluation in the competition score (see Figure 2), and 2) it is a higher threshold than commonly used in previous object detection and segmentation studies (typically 0.5), setting a higher standard for evaluation. Lower thresholds may give the false impression that most of the objects are correctly detected (Table S2 and S3), and that's why modern research studies adopt multiple thresholds in the evaluation functions.

C. Alternative segmentation methods

We note that the segmentation strategy implemented in the five CellProfiler pipelines used for reference segmentation is aligned with widely adopted solutions for nucleus segmentation. For instance, the Cell Tracking Challenge [1], with the participation of experts around the world to test their segmentation and tracking algorithms, reported that 80% of the solutions are based on intensity features, and more specifically 66% of solutions (including the top performing ones) are based on thresholding methodologies, as we do. Also, a recent survey of robust nucleus segmentation lists distance transforms on thresholded images and watershed as a frequent strategy used in microscopy, which is what we adopt in our CellProfiler pipelines. In addition,

both the Cell Tracking Challenge and the nucleus segmentation survey agree that there is no single algorithm that can solve all the cases, and that the best practice is to combine operations depending on the type of images and experimental conditions. This was exactly the approach we used in the CellProfiler pipelines.

Automated parameter optimization is a possibility that could have been used to segment images with our classical algorithm pipelines, however, it still requires creating ground truth or target masks for guiding the search. Creating target masks demands time investment from users, which is not often done in practice. In addition, if target masks are available, running automated parameter optimization for non-machine learning methods may result in high risk of overfitting, yielding solutions that work well only on the few annotated images and do not generalize to the rest of the large un-annotated data. The path towards software that recognizes nuclei without human intervention nor computational expertise is thus less clear for such an approach vs a pre-trained robust nucleus model.

D. Training separate deep learning models

This section describes the design and training of U-Net [4,5] models for each of the five groups defined for evaluation of performance. The goal of these five U-Net models was to evaluate the extent to which standard deep learning architectures can deal with the complexity of the DSB challenge. Clearly, the U-Net architectures used for this experiment are simpler models and have less learning capacity compared to the top three solutions in the final stage of the competition (see Online Methods). However, given that U-Nets are deep learning models widely adopted in the biomedical image analysis community, we consider the result meaningful and of interest.

We used a U-Net architecture with eight convolutional layers, three max pooling layers, and input and output layers with 256x256 pixels, resulting in a total of 7.7 million trainable parameters. This architecture has been used to benchmark nucleus segmentation in fluorescent images [2]. The input images are RGB, as in the dataset of the competition, and the output segmentation maps are also three channels, with probabilities of pixels belonging to background, interior of cells, and boundaries. Then, the first data preparation step was to transform the training target masks as three class segmentation maps. Data augmentation was then configured to produce elastic deformations, 90 degree rotations, flips and illumination variations.

Training was run with similar parameters for all five groups of images after optimizing each using cross validation. Roughly, we run 15 epochs, with 200 gradient updates per epoch, and mini-batches of 10 images per update. Each model was trained in approximately one hour in an NVidia Titan-X GPU. Images from the first stage evaluation set were used for validation of the models and tune learning rates, image scaling factors and other parameters. During testing, each model required different post-processing parameters as well, including the removal of small objects with varying sizes and filtering noise with morphological operations. Finally, the

results reported in the Results section (Figure 1) were obtained by running the model once in the second-state evaluation set.

Overall, the performance of these networks was not particularly satisfactory, given that it required approximately 20 hours of manual work by a data scientist to adapt existing code, configure parameters, validate models and produce the final outputs of the five models, yet, the performance is similar to what a novice can obtain using CellProfiler in five hours (Figure 1). Several reasons contribute to this result, especially the limited learning capacity of U-Nets and the small remaining datasets after splitting the training set in five groups. These two factors make the U-Net models fail, especially with the groups that have few training examples. The large variation from training to testing data is a challenge for these models.

E. Inter-observer variation

Our annotation strategy was designed to maximize the number of annotated objects across a large number of experiments, collecting human segmentation masks for a diverse set of images. Therefore, we prioritized the creation of a large dataset with a wide variety of examples, and decided to tolerate subjective biases and noise introduced by a single annotator. However, the annotation protocol required an expert biologist delineating objects in one image, which was peer-reviewed by other biologist who pointed out missing objects or other mistakes. In this sense, the annotation process was collaborative and involved discussions, revisions, versioning and corrections, mimicking agile software engineering practices to minimize annotation errors.

We decided against consensus-based annotations primarily to reduce the duplicated manual labor of creating masks for each nucleus. Consensus-based masks, which have been widely adopted in previous research, are useful for evaluating pixel-wise precision of segmentations, especially at the edges where most inter-observer variability occurs. Multiple annotators per object are very expensive in the big data regime, and do not necessarily add value for our primary goal of finding all nuclei, given the scale of this data annotation project overall. Thus, we decided that it is more useful to have a large dataset with each image manually annotated by one person than having a small dataset with redundant annotations. As discussed above, peer-review of annotations helped to minimize inconsistencies.

Inter-observer variation is very common in any ground truth definition problem in biology and medicine. It is well known that experts do not agree 100% of the time, thus, research of computational models aims to make more reproducible decisions. Nucleus segmentation is no different, similarly exhibiting inter-observer variation and annotator biases. Our annotations are not real ground truth because we collected manual segmentations from a single observer per object, and their annotations are not independent (we used committee discussions and peer review to fix errors).

For this reason, we estimated inter-observer variation by asking a second annotator to draw manual segmentations for 5 images from the test set, one for each type used in the analysis of results: small fluorescent nuclei, large fluorescent nuclei, purple tissue, pink and purple tissue,

and grayscale tissue. In total, each annotator had to annotate approximately 400 objects. Then, we compared the segmentations produced by the annotators to each other using the intersection-over-union (IoU) metric, as well as the accuracy using F1-score. For comparison, we also included in this analysis the segmentations produced by the top deep learning model as well as the reference CellProfiler segmentations.

The distribution of IoU values between both humans shows relatively high agreement for most of the annotated objects (Figure S3). Most of the objects in the distribution have IoU higher than 0.5, which was the minimum used in the competition score. There are a few outliers with less object overlap, which indicate variations in the edges of small objects (primarily from the purple and grayscale tissue images). Interestingly, the segmentations produced by the top deep learning model follow the same distribution as the inter-human agreement. In terms of object overlap, the deep learning model agrees more often with both humans, than what they agree among themselves (Figure S3, S4, S5). This is because the model tends to fit smooth boundaries close to the edge of nuclei, while humans may introduce noise or hand-drawing biases. In addition, we estimated how many objects a human annotator is likely to miss (IoU < 0.2 in the distribution), and this data indicates that it is approximately 3 objects each 200 annotated masks.

a

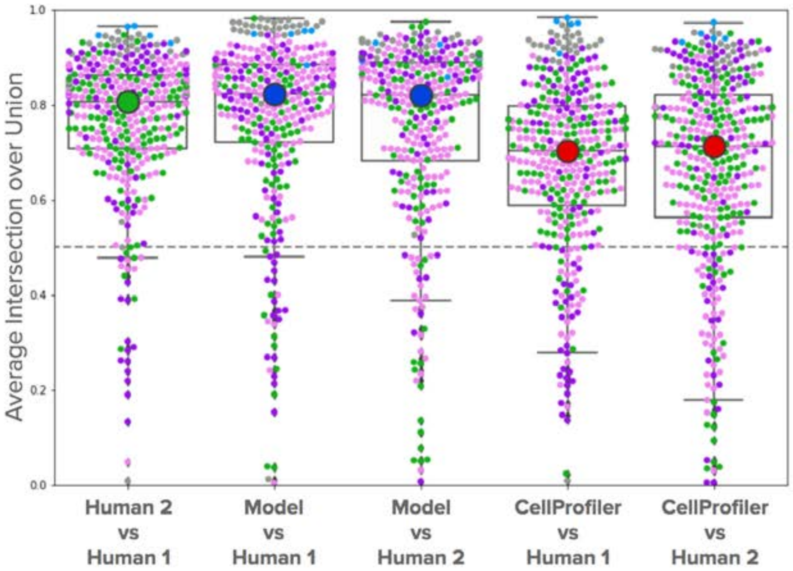

b

Comparing	IoU	F1@0.7
Human 2 vs Human 1	0.806	0.805
Model vs Human 1	0.822	0.801
Model vs Human 2	0.821	0.768
CellProfiler vs Human 1	0.704	0.670
CellProfiler vs Human 2	0.713	0.663

Figure S3. Estimation of inter-observer variability. **a)** each small point in the plot corresponds to one object. The y axis reports the intersection-over-union (IoU) score between compared objects and the x axis reports the pairs of subjects or methods being compared. Large points are the median of all object scores. The color of small points corresponds to the type of image the object comes from (legend in the bottom-right). Notice that nuclei from grayscale tissue images is harder to segment for computational methods, while human annotators generally agree on their masks. Purple objects display disagreement more often, regardless of the pairs being compared. **b)** pairs being compared with measurements of agreement in overlap (IoU) and accuracy (F1-score @ 0.7 IoU). Human annotators (green row)

reach high object overlap agreement, but the top model (blue rows) agrees more often with both humans than what they agree between themselves. However, the model has slightly more disagreement with humans in terms of accuracy, which means the model misses a few objects more frequently than humans do. The CellProfiler reference displays substantial disagreement with humans in terms of overlap and accuracy.

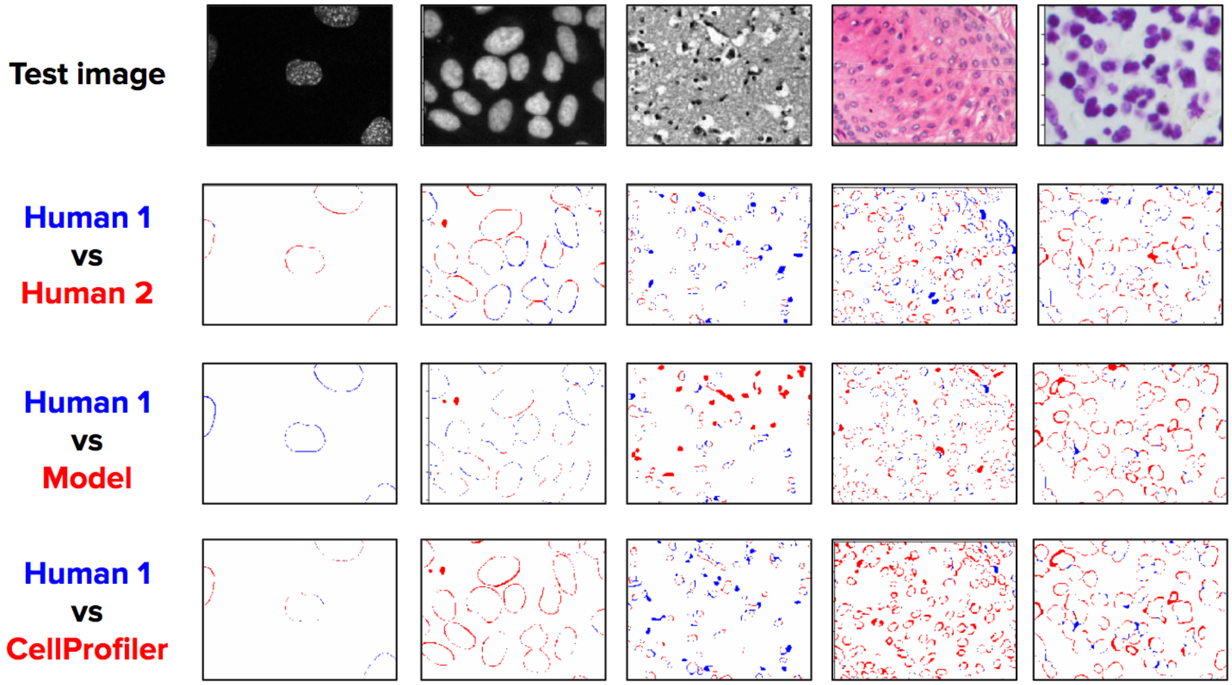

Figure S4. Differences in image annotations in 5 selected image examples. Top row, original images. Next rows, comparison of segmentation between two subjects or methods, one in blue and the other in red. Blue and red objects or outlines indicate annotations introduced by the corresponding observer that do not match the objects or outlines made by the other observer. Ideally, the map should be completely white, which would mean that all annotations are perfectly aligned.

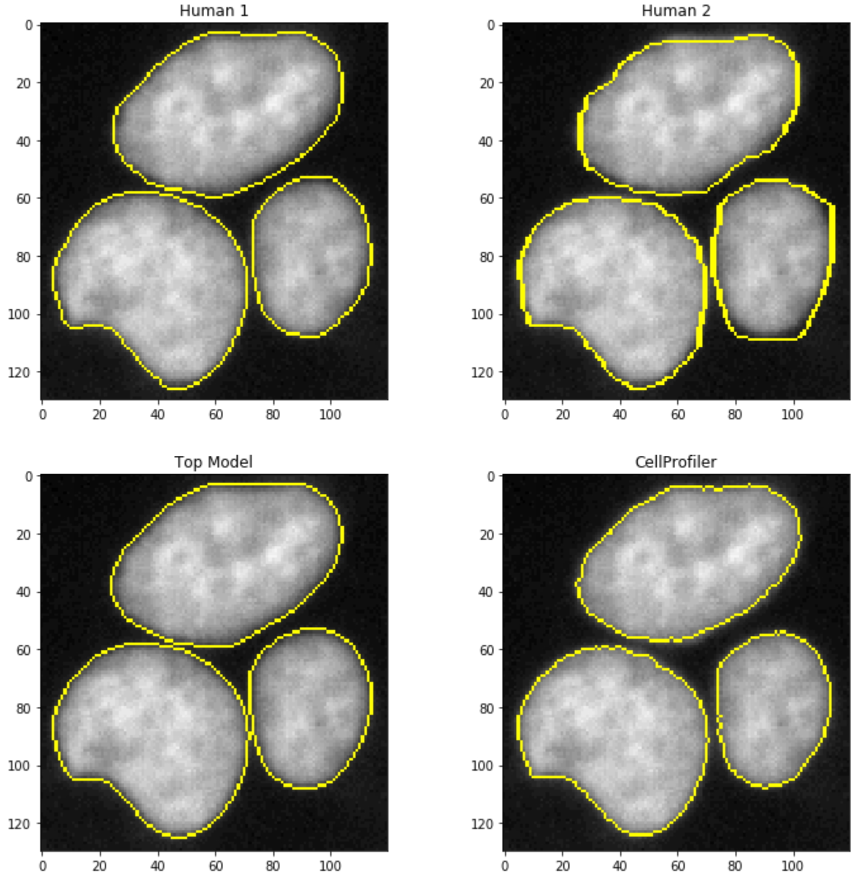

Figure S5. Differences in boundaries between annotators and models. Human annotations introduce subjective noise in the boundaries. The top model learned to produce smooth curves that are close to the edge of the object. This example illustrates how the top model's boundaries can agree more often with both annotators, while humans may disagree on small boundary details. The CellProfiler segmentations, produced with the Watershed algorithm, fit low-level intensity signal that is not as smooth.

F. Error rates

To further explore the differences in performance among the three best-performing solutions, we analyze two types of errors when segmenting nuclei in microscopy images: missed objects and extra objects. These are segmentation artifacts produced when the algorithms fail to interpret the optical signal correctly, for instance, generating a segmentation mask for debris or producing a single segmentation mask for two objects. For most biological applications, these are more serious errors than slight discrepancies in nucleus boundaries, thus, we selected scoring metrics that pay more attention to object-level errors, such as the official competition score and the F1-score (Online Methods), and we also explored the percent missed and percent extra objects as additional useful metrics.

The top three solutions missed fewer objects than the classical segmentation reference. Our CellProfiler pipelines obtain a miss rate of 40% across all second-stage test images, while the

1st, 2nd and 3rd places obtain 22%, 30% and 31% respectively (Figure S6). The top three solutions also provide cleaner segmentations with fewer extra objects, i.e., segmentation masks corresponding to regions without a real object. The CellProfiler reference has a false positive rate of 39% while the top solutions had about 15%. These results indicate that the proposed solutions significantly reduce the number of errors, and thus improve segmentation accuracy.

The percent of errors by image type (Figure S6) is also indicative of which methods are more useful for certain types of images. The best performing solutions display different behaviours in different image types, which suggests the possibility of transferring ideas from one method to another to create a more robust overall method. An example is the category of *Large Fluorescent* images (Figure 2, S6), which contains a total of 48 large objects in the second-stage test set. The solution of the second best participant performs better than the first and third places in reducing both missed and extra objects in this group. This suggests that the multi-scale strategy implemented by the participant in the second place (described below) is more effective in that case.

The results also highlight the challenges in segmenting these images using existing tools such as CellProfiler: the error rates are high despite the pipelines being optimized individually for each of the five image types (Online Methods). The accuracy of segmentation using classical algorithms can probably be improved by creating more specific pipelines for every experiment within each image type class. However, this results in a trade-off between reusability and accuracy, and also increases the time needed to complete the segmentations in practice.

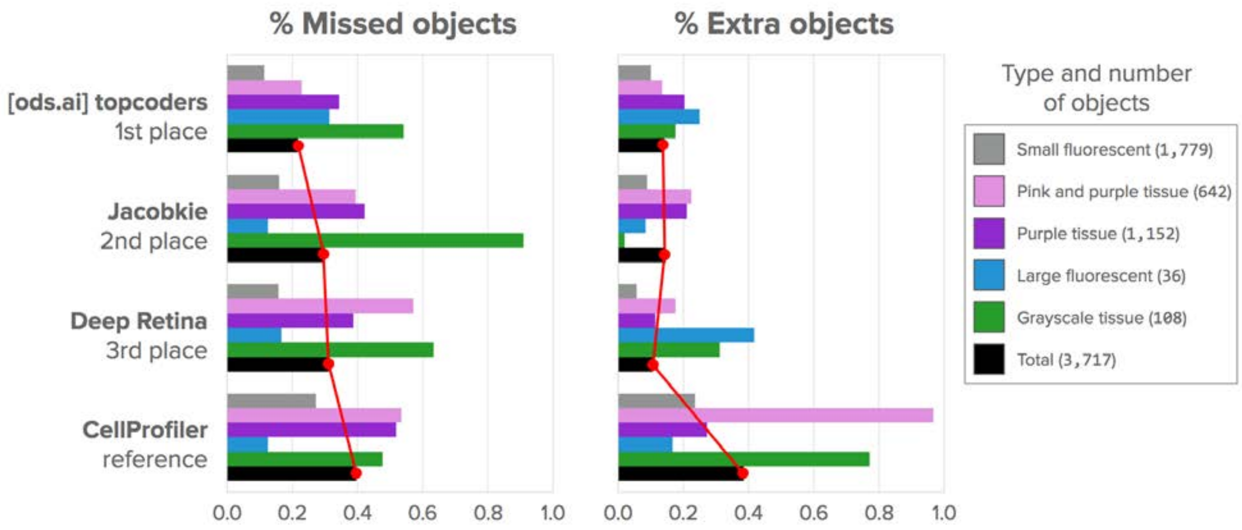

Figure S6. ***Comparison of error rates of the three best performing solutions along with the CellProfiler reference segmentations.*** *The black bars and red dots show global performance for each participant, while color bars indicate performance for each of the visually distinctive groups in the image collection.*

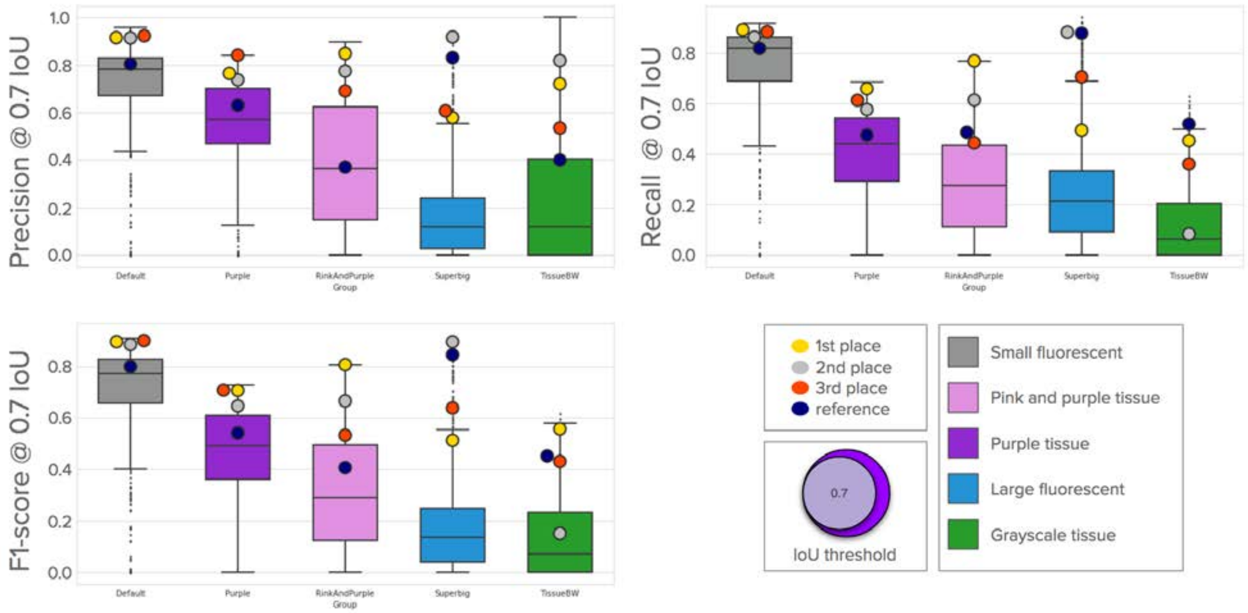

Figure S7. Distribution of precision, recall and F1 scores obtained by all the 739 participants in the second stage evaluation, discriminated by image type. Points in the plot correspond to the top three participants and the CellProfiler segmentation reference. All metrics are measured at the 0.7 intersection over union threshold, which can be interpreted as the requirement that objects have to overlap symmetrically at least with 70% of their area, to be counted as a true positive.

References

1. Ulman, V. *et al.* An objective comparison of cell-tracking algorithms. *Nat. Methods* **14**, 1141–1152 (2017).
2. Caicedo, J. C. *et al.* Evaluation of Deep Learning Strategies for Nucleus Segmentation in Fluorescence Images. *Cytometry A* (2019). doi:10.1002/cyto.a.23863
3. Everingham, M., Van Gool, L., Williams, C. K. I., Winn, J. & Zisserman, A. The Pascal Visual Object Classes (VOC) Challenge. *Int. J. Comput. Vis.* **88**, 303–338 (2010).
4. Ronneberger, O., Fischer, P. & Brox, T. U-net: Convolutional networks for biomedical image segmentation. *Med. Image Comput. Comput. Assist. Interv.* (2015).
5. Falk, T. *et al.* U-Net: deep learning for cell counting, detection, and morphometry. *Nat. Methods* (2018). doi:10.1038/s41592-018-0261-2
