# Supplementary material for: Nucleus segmentation across imaging experiments: the 2018 Data Science Bowl
Source: Nat Methods. 2019 Oct 21;16(12):1247–53. doi: 10.1038/s41592-019-0612-7 (PMC6919559; doi:10.1038/s41592-019-0612-7)
Supplement: Supplementary file 9 — Reporting Summary [file 41592_2019_612_MOESM2_ESM.pdf]

nature research

Corresponding author(s): Anne E. Carpenter

Last updated by author(s): Jul 22, 2019

Reporting Summary

Nature Research wishes to improve the reproducibility of the work that we publish. This form provides structure for consistency and transparency in reporting. For further information on Nature Research policies, see Authors & Referees and the Editorial Policy Checklist.

Statistics

For all statistical analyses, confirm that the following items are present in the figure legend, table legend, main text, or Methods section.

n/a	Confirmed	
☐	☒	The exact sample size (n) for each experimental group/condition, given as a discrete number and unit of measurement
☐	☒	A statement on whether measurements were taken from distinct samples or whether the same sample was measured repeatedly
☒	☐	The statistical test(s) used AND whether they are one- or two-sided<br>*Only common tests should be described solely by name; describe more complex techniques in the Methods section.*
☒	☐	A description of all covariates tested
☒	☐	A description of any assumptions or corrections, such as tests of normality and adjustment for multiple comparisons
☐	☒	A full description of the statistical parameters including central tendency (e.g. means) or other basic estimates (e.g. regression coefficient) AND variation (e.g. standard deviation) or associated estimates of uncertainty (e.g. confidence intervals)
☒	☐	For null hypothesis testing, the test statistic (e.g. F, t, r) with confidence intervals, effect sizes, degrees of freedom and P value noted<br>*Give P values as exact values whenever suitable.*
☒	☐	For Bayesian analysis, information on the choice of priors and Markov chain Monte Carlo settings
☒	☐	For hierarchical and complex designs, identification of the appropriate level for tests and full reporting of outcomes
☒	☐	Estimates of effect sizes (e.g. Cohen's d, Pearson's r), indicating how they were calculated

Our web collection on statistics for biologists contains articles on many of the points above.

Software and code

Policy information about availability of computer code

Data collection	The data collected in this study are microscopy images donated by different contributors. We used CellProfiler, an open source image analysis tool, for normalizing and transforming various image types into a standard format. We also used custom code to create a web-based tool for image annotation, and complemented it with GIMP, an open source photo editor. Finally, we used custom code written in Python to organize the data, generate randomized image names, and reformat manual annotations.
Data analysis	Segmentations generated by participants of the competition were analyzed with custom code written in Python that loads segmentation masks and compares them with masks manually created by experts. This evaluation code generates performance metrics that can be compared from participant to participant to identify the best segmentation model. We used these metrics to analyze types of errors and aggregate statistics of performance. The code to perform our data analysis is publicly available in github at https://github.com/carpenterlab/2019_caicedo_dsb

For manuscripts utilizing custom algorithms or software that are central to the research but not yet described in published literature, software must be made available to editors/reviewers. We strongly encourage code deposition in a community repository (e.g. GitHub). See the Nature Research guidelines for submitting code & software for further information.

Data

Policy information about availability of data

All manuscripts must include a data availability statement. This statement should provide the following information, where applicable:

- Accession codes, unique identifiers, or web links for publicly available datasets
- A list of figures that have associated raw data
- A description of any restrictions on data availability

The image sets used in this study are publicly available with public domain license (CC0), that allows everybody to copy, modify and use for any application, including academic research and commercial ventures. The datasets can be found in https://data.broadinstitute.org/bbbc/BBBC038/

Field-specific reporting

Please select the one below that is the best fit for your research. If you are not sure, read the appropriate sections before making your selection.

☒ Life sciences ☐ Behavioural & social sciences ☐ Ecological, evolutionary & environmental sciences

For a reference copy of the document with all sections, see nature.com/documents/nr-reporting-summary-flat.pdf

Life sciences study design

All studies must disclose on these points even when the disclosure is negative.

Sample size	We collected a total of 841 microscopy images from at least 30 different biological experiments. Each biological experiment may have at least one organism of interest (cell types or tissues). We found a total of 37,333 nuclei that were manually annotated. We did not calculate the size of the sample prior to collecting the dataset. Instead, we aimed to collect as many images as possible while including high experimental experimental variation.
Data exclusions	When preparing the final dataset that was released to the public, we excluded images from certain biological experiments that had a large number of images. In other words, we avoided over-representing images from a single experiment and promoted the diversity of images.
Replication	The image dataset represents 30 biological experiments from different labs and different experimental conditions. We collected them such that different types of experiments may have several instances, e.g. several tissue experiments, several high-throughput experiments, and so on. We do not have exact replicates, but our goal was to incorporate similar experiments with technical variation coming from different laboratories.
Randomization	The image sets in three groups: 1) a training set, fully accessible to everybody during the competition, 2) a validation set, with public images and private segmentation masks, and 3) a test set, with public images only. The training and validation set come from 16 biological experiments, while the test set comes from 15 different biological experiments not used in any of the previous tests. With this partition, we followed the best data science practices for validation of segmentation models that can generalize to new samples beyond the training set.
Blinding	Participants of the competition were blind of the manual annotations created by experts on the validation and test sets. The worked on segmentation models using manual annotations in the training set, and they did not know which images were going to be evaluated and what the correct answer was supposed to be.

Reporting for specific materials, systems and methods

We require information from authors about some types of materials, experimental systems and methods used in many studies. Here, indicate whether each material, system or method listed is relevant to your study. If you are not sure if a list item applies to your research, read the appropriate section before selecting a response.

Materials & experimental systems

n/a	Involved in the study
☒	☐ Antibodies
☒	☐ Eukaryotic cell lines
☒	☐ Palaeontology
☒	☐ Animals and other organisms
☒	☐ Human research participants
☒	☐ Clinical data

Methods

n/a	Involved in the study
☒	☐ ChIP-seq
☒	☐ Flow cytometry
☒	☐ MRI-based neuroimaging
