# Supplementary material for: Nucleus segmentation across imaging experiments: the 2018 Data Science Bowl
Source: Nat Methods. 2019 Oct 21;16(12):1247–53. doi: 10.1038/s41592-019-0612-7 (PMC6919559; doi:10.1038/s41592-019-0612-7)
Supplement: Supplementary file 10 — Supplementary Notes 1–7. [file 41592_2019_612_MOESM3_ESM.pdf]

Supplementary Notes

Note 1. Dataset statistics

In the context of this dataset, an experiment is defined as the biological study of a specimen, which includes the physical preparation of a sample for microscopy evaluation, the development of the imaging assay, equipment configuration, and image acquisition. Normally, a biological experiment is conducted in a facility, by a group of researchers, using specific equipment in a bounded time schedule.

The description of all image sets is reported in Supplementary Table 1, which contains quantitative information about images, including resolution, total number of nuclei, SNR and contrast (computed according to Ulman et al. (2017) [1]. It also contains qualitative information such as cell type and visual group assigned based on the optical appearance. In summary, the full dataset comprises 37,333 manually annotated nuclei, 841 images, 31 experiments, 15 cell types, 12 image resolutions, and 5 visually similar groups. The average SNR in all experiments is 2.488 with a standard deviation of 1.697. The 31 experiments were assigned to two partitions, 16 experiments for training and first stage evaluation, the remaining 15 experiments for the second stage evaluation.

We collected only those metadata parameters that were available to all experiments. Not all experiments have detailed metadata information about their development because images were donated from labs all around the world: some of them had metadata available, some others did not, some of them had confidential information that cannot be shared, some others were already publicly available on the web and did not report these properties. Our team of expert biologists curated and annotated the image collection to the best of their ability, and included images that can add value even without complete descriptions.

The most challenging aspect of the dataset is the large experimental variation. For our purposes, we treated the dataset variables as unknown confounders, except for the source laboratory, which is a proxy variable to all other details. We selected a few images from large experiments to avoid over-representation of samples with the same technical variations. In some cases a random sample was used, in other cases, manual selection was performed. We did not balance the dataset based on the five visually distinctive groups of images, as our goal was to introduce technical and experimental diversity to simulate the real world variations that a widely applicable nucleus segmentation method would find in the wild.

Note 2. Description of evaluation metrics

The Intersection-over-Union (IoU) metric is used to estimate object coverage and detect correctly segmented nuclei. There are several types of errors that can be measured at one IoU threshold, including merged, split, missed and extra objects [2] following this procedure: given N

segmented objects and M target objects, we compute the $N \times M$ matrix of IoU scores. Ideally, this is a square and diagonal matrix. However, with extra objects the matrix will have more rows, and with missed objects it will have less rows. Over-segmented objects appear as extra rows with at least two non-zero entries in the corresponding target mask column. We keep the estimated segmentation with maximum IoU for each target mask (only if max-IoU is above a threshold) and the remaining pieces are counted as false positive objects.

The official competition metric looks at true positives, false positives and false negatives at multiple IoU thresholds and computes a precision score as originally suggested by Everingham et al. [3] for semantic segmentation. These scores are averaged across all images and all thresholds. In the study presented in this paper we also computed the F1-score, which is defined as the harmonic average of precision and recall, and is used to compute the accuracy of a test. We use F1-scores to interpret the accuracy results in the context of nucleus segmentation given that there is only one class of objects to be segmented, and the score indicates whether it's correctly segmented or not [2].

All scores are normalized by image, which means that if an image contains significantly more objects than others, this will not affect the results and will make scores comparable from image to image. The scores are not normalized by dataset, image type or experiment. These variables are ignored when computing our aggregated scores for simplicity. During the competition we did not assign more value to certain types of images, all were treated equally. However, in our analysis of the results, we decomposed scores by image type because we observed that this variable drives an important part of the error. This can be observed in Figures 1c, 2a, and 3.

In several analyses run after the competition ended, we used a single IoU threshold to compare performance, estimate error rates, and interpret the results in a more intuitive way. We chose IoU=0.7 as our reference cut-off for all analyses that require a single threshold in this paper. We made this choice for two reasons: 1) it is a central point in the range of thresholds used for evaluation in the competition score (see Figure 2), and 2) it is a higher threshold than commonly used in previous object detection and segmentation studies (typically 0.5), setting a higher standard for evaluation. Lower thresholds may give the false impression that most of the objects are correctly detected (Table S2 and S3), and that's why modern research studies adopt multiple thresholds in the evaluation functions.

Note 3. Alternative segmentation methods

We note that the segmentation strategy implemented in the five CellProfiler pipelines used for reference segmentation is aligned with widely adopted solutions for nucleus segmentation. For instance, the Cell Tracking Challenge [1], with the participation of experts around the world to test their segmentation and tracking algorithms, reported that 80% of the solutions are based on intensity features, and more specifically 66% of solutions (including the top performing ones) are based on thresholding methodologies, as we do. Also, a recent survey of robust nucleus segmentation lists distance transforms on thresholded images and watershed as a frequent strategy used in microscopy, which is what we adopt in our CellProfiler pipelines. In addition,

both the Cell Tracking Challenge and the nucleus segmentation survey agree that there is no single algorithm that can solve all the cases, and that the best practice is to combine operations depending on the type of images and experimental conditions. This was exactly the approach we used in the CellProfiler pipelines.

Automated parameter optimization is a possibility that could have been used to segment images with our classical algorithm pipelines, however, it still requires creating ground truth or target masks for guiding the search. Creating target masks demands time investment from users, which is not often done in practice. In addition, if target masks are available, running automated parameter optimization for non-machine learning methods may result in high risk of overfitting, yielding solutions that work well only on the few annotated images and do not generalize to the rest of the large un-annotated data. The path towards software that recognizes nuclei without human intervention nor computational expertise is thus less clear for such an approach vs a pre-trained robust nucleus model.

Note 4. Training separate deep learning models

This section describes the design and training of U-Net [4,5] models for each of the five groups defined for evaluation of performance. The goal of these five U-Net models was to evaluate the extent to which standard deep learning architectures can deal with the complexity of the DSB challenge. Clearly, the U-Net architectures used for this experiment are simpler models and have less learning capacity compared to the top three solutions in the final stage of the competition (see Online Methods). However, given that U-Nets are deep learning models widely adopted in the biomedical image analysis community, we consider the result meaningful and of interest.

We used a U-Net architecture with eight convolutional layers, three max pooling layers, and input and output layers with 256x256 pixels, resulting in a total of 7.7 million trainable parameters. This architecture has been used to benchmark nucleus segmentation in fluorescent images [2]. The input images are RGB, as in the dataset of the competition, and the output segmentation maps are also three channels, with probabilities of pixels belonging to background, interior of cells, and boundaries. Then, the first data preparation step was to transform the training target masks as three class segmentation maps. Data augmentation was then configured to produce elastic deformations, 90 degree rotations, flips and illumination variations.

Training was run with similar parameters for all five groups of images after optimizing each using cross validation. Roughly, we run 15 epochs, with 200 gradient updates per epoch, and mini-batches of 10 images per update. Each model was trained in approximately one hour in an NVidia Titan-X GPU. Images from the first stage evaluation set were used for validation of the models and tune learning rates, image scaling factors and other parameters. During testing, each model required different post-processing parameters as well, including the removal of small objects with varying sizes and filtering noise with morphological operations. Finally, the

results reported in the Results section (Figure 1) were obtained by running the model once in the second-state evaluation set.

Overall, the performance of these networks was not particularly satisfactory, given that it required approximately 20 hours of manual work by a data scientist to adapt existing code, configure parameters, validate models and produce the final outputs of the five models, yet, the performance is similar to what a novice can obtain using CellProfiler in five hours (Figure 1). Several reasons contribute to this result, especially the limited learning capacity of U-Nets and the small remaining datasets after splitting the training set in five groups. These two factors make the U-Net models fail, especially with the groups that have few training examples. The large variation from training to testing data is a challenge for these models.

Note 5. Inter-observer variation

Our annotation strategy was designed to maximize the number of annotated objects across a large number of experiments, collecting human segmentation masks for a diverse set of images. Therefore, we prioritized the creation of a large dataset with a wide variety of examples, and decided to tolerate subjective biases and noise introduced by a single annotator. However, the annotation protocol required an expert biologist delineating objects in one image, which was peer-reviewed by other biologist who pointed out missing objects or other mistakes. In this sense, the annotation process was collaborative and involved discussions, revisions, versioning and corrections, mimicking agile software engineering practices to minimize annotation errors.

We decided against consensus-based annotations primarily to reduce the duplicated manual labor of creating masks for each nucleus. Consensus-based masks, which have been widely adopted in previous research, are useful for evaluating pixel-wise precision of segmentations, especially at the edges where most inter-observer variability occurs. Multiple annotators per object are very expensive in the big data regime, and do not necessarily add value for our primary goal of finding all nuclei, given the scale of this data annotation project overall. Thus, we decided that it is more useful to have a large dataset with each image manually annotated by one person than having a small dataset with redundant annotations. As discussed above, peer-review of annotations helped to minimize inconsistencies.

Inter-observer variation is very common in any ground truth definition problem in biology and medicine. It is well known that experts do not agree 100% of the time, thus, research of computational models aims to make more reproducible decisions. Nucleus segmentation is no different, similarly exhibiting inter-observer variation and annotator biases. Our annotations are not real ground truth because we collected manual segmentations from a single observer per object, and their annotations are not independent (we used committee discussions and peer review to fix errors).

For this reason, we estimated inter-observer variation by asking a second annotator to draw manual segmentations for 5 images from the test set, one for each type used in the analysis of results: small fluorescent nuclei, large fluorescent nuclei, purple tissue, pink and purple tissue,

and grayscale tissue. In total, each annotator had to annotate approximately 400 objects. Then, we compared the segmentations produced by the annotators to each other using the intersection-over-union (IoU) metric, as well as the accuracy using F1-score. For comparison, we also included in this analysis the segmentations produced by the top deep learning model as well as the reference CellProfiler segmentations.

The distribution of IoU values between both humans shows relatively high agreement for most of the annotated objects (Figure S3). Most of the objects in the distribution have IoU higher than 0.5, which was the minimum used in the competition score. There are a few outliers with less object overlap, which indicate variations in the edges of small objects (primarily from the purple and grayscale tissue images). Interestingly, the segmentations produced by the top deep learning model follow the same distribution as the inter-human agreement. In terms of object overlap, the deep learning model agrees more often with both humans, than what they agree among themselves (Figure S3, S4, S5). This is because the model tends to fit smooth boundaries close to the edge of nuclei, while humans may introduce noise or hand-drawing biases. In addition, we estimated how many objects a human annotator is likely to miss (IoU < 0.2 in the distribution), and this data indicates that it is approximately 3 objects each 200 annotated masks.

Note 6. Error rates

To further explore the differences in performance among the three best-performing solutions, we analyze two types of errors when segmenting nuclei in microscopy images: missed objects and extra objects. These are segmentation artifacts produced when the algorithms fail to interpret the optical signal correctly, for instance, generating a segmentation mask for debris or producing a single segmentation mask for two objects. For most biological applications, these are more serious errors than slight discrepancies in nucleus boundaries, thus, we selected scoring metrics that pay more attention to object-level errors, such as the official competition score and the F1-score (Online Methods), and we also explored the percent missed and percent extra objects as additional useful metrics.

The top three solutions missed fewer objects than the classical segmentation reference. Our CellProfiler pipelines obtain a miss rate of 40% across all second-stage test images, while the 1st, 2nd and 3rd places obtain 22%, 30% and 31% respectively (Figure S6). The top three solutions also provide cleaner segmentations with fewer extra objects, i.e., segmentation masks corresponding to regions without a real object. The CellProfiler reference has a false positive rate of 39% while the top solutions had about 15%. These results indicate that the proposed solutions significantly reduce the number of errors, and thus improve segmentation accuracy.

The percent of errors by image type (Figure S6) is also indicative of which methods are more useful for certain types of images. The best performing solutions display different behaviours in different image types, which suggests the possibility of transferring ideas from one method to another to create a more robust overall method. An example is the category of *Large Fluorescent* images (Figure 2, S6), which contains a total of 48 large objects in the second-

stage test set. The solution of the second best participant performs better than the first and third places in reducing both missed and extra objects in this group. This suggests that the multi-scale strategy implemented by the participant in the second place (described below) is more effective in that case.

The results also highlight the challenges in segmenting these images using existing tools such as CellProfiler: the error rates are high despite the pipelines being optimized individually for each of the five image types (Online Methods). The accuracy of segmentation using classical algorithms can probably be improved by creating more specific pipelines for every experiment within each image type class. However, this results in a trade-off between reusability and accuracy, and also increases the time needed to complete the segmentations in practice.

Note 7. Additional links

The following is a list of links to discussions in the public Kaggle forum where participants of the competition shared information about their models and organizers provided additional details about the challenge. This compilation of links supplements other discussions presented in the paper.

- ***Credits for images used in the challenge***. A thank you note to all who donated images to create the dataset for the Data Science Bowl 2018. https://www.kaggle.com/c/data-science-bowl-2018/discussion/54759
- ***Annotation strategies used to generate nucleus masks***. Explanations of how the manual annotations were generated. https://www.kaggle.com/c/data-science-bowl-2018/discussion/54900
- ***Annotation protocol using GIMP***. Description of the annotation protocol used to create the second stage evaluation reference masks. https://www.kaggle.com/c/data-science-bowl-2018/discussion/48347
- ***Description of the evaluation metric and protocol***. Details of how evaluation scores were computed in the competition and description of the file format for submissions. https://www.kaggle.com/c/data-science-bowl-2018/overview/evaluation
- ***First place solution***. Details of the model created by the winning team. https://www.kaggle.com/c/data-science-bowl-2018/discussion/54741
- ***Second place solution***. Details of the model created by the second-place team. https://www.kaggle.com/c/data-science-bowl-2018/discussion/61170
- ***Third place solution***. Details of the model created by the third-place team. https://www.kaggle.com/c/data-science-bowl-2018/discussion/56393

References

1. Ulman, V. *et al.* An objective comparison of cell-tracking algorithms. *Nat. Methods* **14**, 1141–1152 (2017).
2. Caicedo, J. C. *et al.* Evaluation of Deep Learning Strategies for Nucleus Segmentation in Fluorescence Images. *Cytometry A* (2019). doi:10.1002/cyto.a.23863
3. Everingham, M., Van Gool, L., Williams, C. K. I., Winn, J. & Zisserman, A. The Pascal Visual Object Classes (VOC) Challenge. *Int. J. Comput. Vis.* **88**, 303–338 (2010).
4. Ronneberger, O., Fischer, P. & Brox, T. U-net: Convolutional networks for biomedical image segmentation. *Med. Image Comput. Comput. Assist. Interv.* (2015).
5. Falk, T. *et al.* U-Net: deep learning for cell counting, detection, and morphometry. *Nat. Methods* (2018). doi:10.1038/s41592-018-0261-2
